# Long-Term Biogeochemical Stabilization of Carbonate-Bearing Pb–Zn–Cu Mine Tailings by Amendment-Assisted Phytostabilization

**DOI:** 10.1021/acs.est.6c08282

**Published:** 2026-10-15

**Authors:** Mariam Al-Lami, Genevieve Sutton, Kwame Awuah-Offei, Lana Alagha, Joel Burken

**Affiliations:** † Department of Civil, Architectural and Environmental Engineering, Missouri University of Science and Technology, Rolla, Missouri 65409, United States; ‡ 51526 The Doe Run Company, St. Louis, Missouri 63146, United States; § Department of Mining & Explosives Engineering, 14717 Missouri University of Science & Technology, Rolla, Missouri 65409, United States

**Keywords:** arbuscular mycorrhizal fungi, biosolids, biochar, dissolved metals, sequential extraction, metal lability, organic carbon accumulation, soil development

## Abstract

Legacy Pb–Zn–Cu mine tailings can remain barren and geochemically reactive for decades. Although amendment-assisted revegetation can establish short-term plant growth, sustained phytostabilization requires metal attenuation and soil-like development over years after amendment-derived nutrients and organic matter decline. We evaluated seven-year biogeochemical stabilization of carbonate-bearing tailings planted with the native legume *Dalea purpurea*. Biosolids (BS), biochar (BC), and arbuscular mycorrhizal fungi (AMF) were incorporated into the upper 8 cm, while the underlying 12 cm remained unamended. Tailings pore water was monitored annually, and year-seven cores were analyzed by depth for organic carbon, PLFA-based microbial indicators, aqua-regia-extractable metals, sequential metal fractionation, and X-ray diffraction. Biosolids initially increased EC, DOC, DN, and dissolved Pb, Zn, and Cu, whereas BC- and/or AMF-containing treatments showed greater long-term reductions in dissolved metal concentrations. AMF-inoculated treatments increased organic carbon and microbial biomass indicators below the amended layer, while deeper root penetration was also observed, consistent with profile-scale biological development. Sequential extraction showed metal-specific repartitioning: Pb was primarily carbonate- and Fe/Mn oxide-associated; Zn remained partly carbonate-linked; and Cu shifted toward organic/sulfide-associated and residual pools, consistent with lower relative lability. These findings show that surface-applied amendment and planting strategies can promote coupled biological and geochemical stabilization in initially unamended deeper tailings layers, supporting ecosystem restoration beyond short-term surface revegetation.

## Graphical abstract

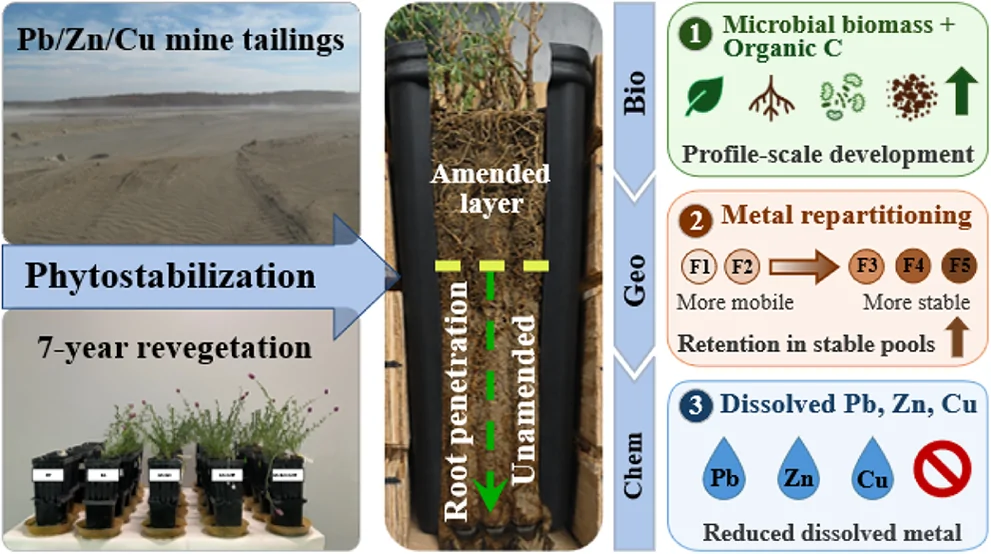

## 1 Introduction

Legacy mine tailings remain persistent sources of metal exposure because they are not inert waste deposits but reactive geochemical systems. Their low organic matter, poor structure, limited nutrient availability, and elevated metal concentrations restrict plant and microbial establishment while leaving tailings surfaces vulnerable to wind dispersion, runoff, and pore-water transport.^1,2^ In Pb–Zn–Cu tailings, risk is governed not only by total metal concentrations but also by the distribution of metals among pore water, readily labile pools, reactive carbonate- and Fe/Mn oxide-associated phases, organic/sulfide-associated pools, and more stable residual fractions.^3–5^ This distinction is particularly important for carbonate-bearing tailings, where circumneutral to alkaline pH can suppress immediate metal solubility, but longer-term mobility may still be influenced by carbonate weathering, dissolved organic carbon, ionic strength, and redox-sensitive mineral phases.^6–8^

Amendment-assisted phytostabilization offers a practical alternative to excavation or engineered capping for large mine-waste areas, but its success is often evaluated too narrowly. Recent restoration approaches increasingly emphasize nature-based strategies that combine contaminant stabilization with development of ecological functions, including carbon cycling and microbial development.^9,10^ Field-based phytomanagement studies have demonstrated that combined amendment and native plant strategies can improve restoration outcomes in metal-contaminated mine residuals.^11,12^ Vegetation can reduce surface erosion, improve surface stability, and initiate organic carbon deposition; however, plant survival or biomass alone does not demonstrate geochemical stabilization or development of broader biological and biogeochemical functions.^13,14^ A more rigorous assessment requires evidence that amendment–plant systems reduce metal lability, promote retention in less mobile solid-phase pools, and support the development of biological and biogeochemical functions. Biosolids (BS) can improve tailings fertility, organic matter content, microbial activity, and metal retention through organic matter inputs and sorption/complexation processes.^15^ However, their long-term effectiveness may be limited by decomposition of labile organic matter, redistribution of nutrients, and potential contributions of dissolved organic ligands, salts, and metal inputs that influence pore-water chemistry and metal mobility.^16,17^ Biochar (BC) and arbuscular mycorrhizal fungi (AMF) may complement biosolids by increasing sorptive capacity, improving nutrient retention, promoting aggregation, and supporting root- and mycorrhizosphere-mediated carbon inputs and microbial development.^18,19^ Recent studies have shown that integrated amendment mixtures containing biochar, organic amendments, and microbial inocula can improve plant establishment, modify tailings chemistry, and influence microbial communities in mine rehabilitation settings.^18,20^

Despite these potential benefits, the time-resolved and depth-dependent coupling between biological development and geochemical stabilization in amended tailings remains insufficiently explored. Previous studies have examined improvements in vegetation establishment, metal bioavailability, microbial indicators, or microbial community structure during tailings and mine-soil rehabilitation.^21–25^ Long-term rehabilitation studies have further demonstrated progressive changes in tailings chemistry, organic matter characteristics, and microbial communities following revegetation.^26^ However, the persistence and extension of coupled biological and geochemical responses into initially unamended tailings layers remain poorly resolved, particularly when considering long-term changes in pore-water chemistry alongside solid-phase metal partitioning, organic carbon content, and microbial characteristics. This limitation is important because sustained phytostabilization requires not only reduced metal mobility but also development of functional tailings properties beyond the amended surface layer. Biological amendments such as AMF may contribute to belowground development through root-associated processes and microbial activity, potentially facilitating carbon accumulation and microbial development beyond the amended surface layer. The contribution of these processes to sustained geochemical stabilization in initially unamended deeper tailings, however, remains uncertain.

Here, we evaluated the long-term biogeochemical stabilization of carbonate-bearing Pb–Zn–Cu mine tailings under amendment-assisted revegetation with purple prairie clover (*Dalea purpurea*), a native prairie legume species. Biosolids, biochar, and arbuscular mycorrhizal fungi were applied only to the upper tailings layer, while the underlying tailings remained unamended, providing a depth-resolved framework for evaluating treatment-associated responses beyond the zone of direct amendment incorporation. Over seven years, tailings pore-water was monitored annually for pH, electrical conductivity, dissolved organic carbon, dissolved nitrogen, and dissolved Pb, Zn, and Cu. At the end of the seventh growth season, tailings cores were analyzed for organic carbon, phospholipid fatty acid (PLFA)-based microbial indicators, aqua-regia-extractable metals, sequentially extracted Pb, Zn, Cu, Fe, and Mn fractions, and mineralogy by X-ray diffraction (XRD). We hypothesized that long-term amendment-assisted revegetation would: (i) alter pore-water chemistry and dissolved metal concentrations; (ii) promote organic carbon accumulation and microbial development within and below the amended layer; and (iii) shift Pb, Zn, and Cu among operationally defined fractions in a metal- and depth-dependent manner. Together, this framework evaluates phytostabilization as a coupled aqueous, solid-phase, and biological process rather than vegetation establishment alone.

## 2 Materials and Methods

### 2.1 Mine Tailings, Amendments, and Plant Species

Mine tailings were collected from a Pb–Zn–Cu tailings impoundment in the Viburnum Trend mining district, Missouri, USA (37°42′09.0″N, 91°06′18.4″W). The site and tailings material have been described previously in studies evaluating amendment-assisted revegetation and phytomanagement of the same Pb–Zn–Cu mine residuals.^27–29^ The tailings were collected from the upper 0–30 cm layer, air-dried, crushed, homogenized, and passed through a 2 mm sieve prior to use. The tailings are carbonate-bearing, finely textured mine residues associated with Pb–Zn–Cu mineralization in the Viburnum Trend.^30^ These characteristics are consistent with the carbonate-hosted Mississippi Valley-type geologic setting, where Pb–Zn–Cu mineralization occurs in carbonate host rocks.^31–33^ Tailings from the district are commonly dolomite-rich and may contain residual ore minerals after beneficiation.^34^

Biosolids used in this study were collected from the Viburnum city sanitary lagoons, located approximately 1 km northwest of the tailings impoundment (37°42′36.8″N, 91°07′04.6″W). These biosolids had accumulated in the lagoons for approximately 40 years and contained elevated metal concentrations (Table 1). The collected material was transported to the greenhouse, dewatered, and dried to approximately 35% solids before amendment application. Pine wood-derived biochar was obtained from a local source near the tailings site. Characteristics of the tailings and amendments are summarized in Table 1.

**Table 1:** Physicochemical Characterization of Mine Tailings and Amendments Used in This Study^d^

Parameter	Mine tailings (untreated)	Background levels^a^	Biosolids (BS)	Biochar (BC)
pH	7.6	>6.1^b^	4.98	8.61
Neutralizable acidity (meq 100 g^–1^)	0	–	–	–
EC (mS cm^–1^)	1.2	–	8.13	0.56
CEC (cmolc kg^–1^)	3.7	5–25^c^	21.6	25.1
OM (%)	0.2	–	46.3	–
Total N (%)	0.06	–	2.66	0.25
Total P (%)	0.009	–	0.938	0.041
NH_4_^+^–N (mg kg^–1^)	0.45	–	5.05	0.14
NO_3_^–^–N (mg kg^–1^)	3.3	–	1,256	0.48
Extractable K (mg kg^–1^)	31.0	–	–	–
Extractable Ca (mg kg^–1^)	418	–	–	–
Extractable Mg (mg kg^–1^)	188.5	–	–	–
**Major elements (%)**				
Total K	0.031	–	0.096	0.383
Total Ca	15.65	–	4.16	2.13
Total Mg	10.3	–	0.993	0.072
**Total metals** **(mg kg** ^**–1**^ **)**				
Pb	4,086.2	20^a^	3,409.8	2.26
Zn	993.8	49^a^	2,708.2	6.3
Cu	557.9	13^a^	1,958.5	2.3
Fe	23,627.4	–	24,886	145
Mn	1,352.9	–	468	557
Al	1,234.2	–	–	–
Ni	75.3	14	–	–
Cd	13.9	<1	–	–

a Background concentration for metals in Missouri soils.^36^

b Summary of soil fertility status in Missouri.^37^

c Scrivner and Cooper 1985.^38^

d Values represent characterization results from composite samples prepared from homogenized bulk materials; analytical methods are described in Section 2.2.

A native arbuscular mycorrhizal fungal inoculum originating from high-nutrient prairie soils in Kansas and Missouri was used as the biological amendment. The inoculum was obtained from MycoBloom (Lawrence, KS, USA) and contained spores, hyphal mycelium, and colonized root fragments from multiple AMF species (Supporting Information). Purple prairie clover (*Dalea purpurea*) seeds were obtained from Prairie Moon Nursery and inoculated with compatible rhizobium bacteria before planting.

### 2.2 Tailings and Amendment Characterization

Tailings and amendment materials were characterized by the Soil and Plant Testing Laboratory at the University of Missouri, Columbia, MO, following the laboratory’s standard procedures.^35^ Prior to characterization, materials were homogenized, and subsamples collected from multiple locations within each bulk material were pooled to form composite samples. Table 1 values represent characterization results obtained from composite samples.

For tailings, pH and electrical conductivity (EC) were determined using a 1:1 soil-to-water extraction following MU soil testing procedures. For biosolids and biochar, pH was measured directly in a distilled-water saturated paste after 30 min equilibration, and EC was determined from the filtered saturated paste extract following compost analysis procedures. Organic matter was determined by loss on ignition. Exchangeable K, Ca, Mg, and Na were measured using 1 N ammonium acetate extraction buffered at pH 7.0, and cation exchange capacity (CEC) was calculated by summation of exchangeable base cations and neutralizable acidity. Total nitrogen and phosphorus were determined by Kjeldahl digestion followed by flow injection analysis, and total Pb, Zn, Cu, Fe, Mn, and other elemental concentrations were determined following EPA Method 3050B digestion and ICP-OES analysis.

### 2.3 Long-Term Pot Experiment

A seven-year greenhouse pot experiment was established to determine whether surface-applied amendments could promote long-term biogeochemical stabilization of carbonate-bearing Pb–Zn–Cu tailings and stimulate soil development below the amended zone. Deep, tapered square plastic treepots with approximate dimensions of 10 cm width × 10 cm length × 25 cm depth were used. Pots were filled to approximately 20 cm depth, leaving 5 cm headspace. Each pot was packed with a stratified tailings profile consisting of approximately 1.4 kg of unamended tailings in the lower 12 cm and approximately 1.0 kg of untreated or amended tailings in the upper 8 cm. Amendments were incorporated only into the upper layer, while the lower layer remained unamended to assess whether surface treatment effects extended downward through root development, pore-water movement, and associated biogeochemical processes (Figure 1).

**Figure 1:**
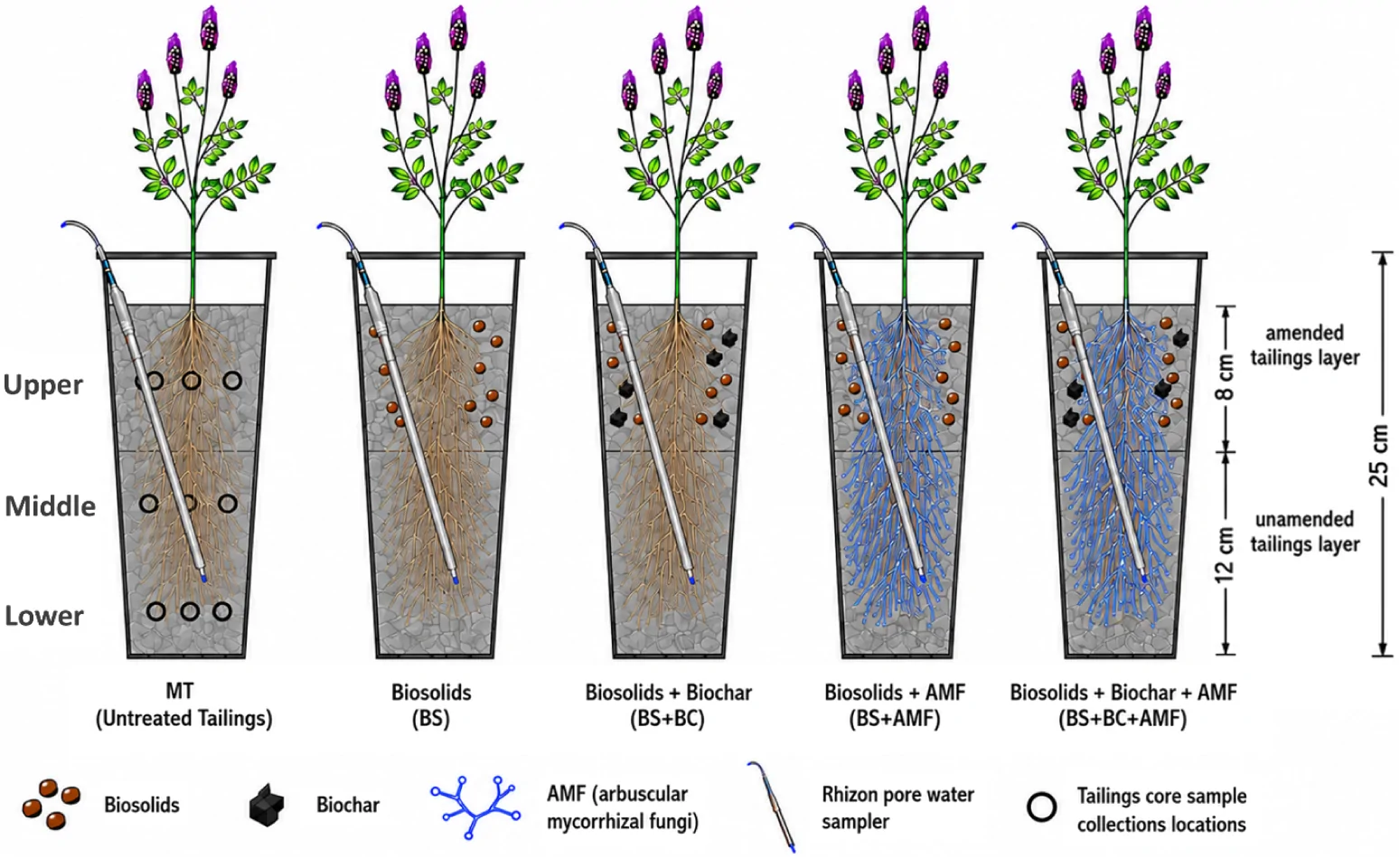
Experimental design schematic for the seven-year pot experiment with *Dalea purpurea* grown in Pb–Zn–Cu mine tailings. Amendments and AMF were applied only to the upper tailings layer; the lower layer remained unamended. The figure was prepared from an original experimental design schematic and refined using AI-assisted visual tools; the final figure was reviewed and edited by the authors for scientific accuracy.

Five treatments were prepared: untreated mine tailings (MT), tailings+biosolids (BS), tailings + biosolids + biochar (BS+BC), tailings + biosolids + arbuscular mycorrhizal fungi (BS+AMF), and tailings + biosolids + biochar + arbuscular mycorrhizal fungi (BS+BC+AMF) (Figure 1). Biosolids, BC, and AMF were applied to the upper amended layer at 3%, 2%, and 5% (w/w), respectively. Nonmycorrhizal treatments received an equivalent amount of sterilized AMF inoculum in the upper layer to maintain comparable inoculum-carrier inputs across treatments. The amendments and application rates were selected based on previous studies using the same Pb–Zn–Cu tailings.^27,29,39^

The treatment combinations were designed to evaluate complementary amendment functions during long-term revegetation. BS was used as the primary organic matter and nutrient amendment, BC was included as a locally available carbonaceous and sorptive amendment, and AMF was included as a biological amendment. The combined BS+BC+AMF treatment was included to test whether these functions could act together to promote more sustained revegetation and biogeochemical stabilization than BS alone. The MT treatment received no amendment and served as the unamended control. Each treatment was replicated five times. The experiment was arranged as a completely randomized design, and pot positions were periodically rerandomized within the greenhouse.

Purple prairie clover (*Dalea purpurea*) was selected as the native revegetation species based on its prairie origin, leguminous growth habit, previous performance in screening studies with the same Pb–Zn–Cu tailings,^39^ and relevance to revegetation of nutrient-poor mine tailings and AMF-associated native prairie restoration.^40^ Seeds were sown in the upper tailings layer after treatment preparation, and plants were maintained under greenhouse conditions at Missouri University of Science and Technology, Rolla, MO, for a seven-year growth period. Plants were grown under average night/day temperatures of 20/27 °C, approximately 80% relative humidity, and a 16 h photoperiod. Pots were watered three times per week with deionized (DI) water to maintain approximately 70% of field capacity, and shallow trays were placed beneath each pot to collect leachate.

### 2.4 Tailings Pore-Water Collection and Analysis

For annual tailings pore-water (TPW) collection, one Rhizon MOM soil moisture sampler (10 cm porous membrane length, 2.5 mm diameter, 0.15 μm pore size; Royal Eijkelkamp, The Netherlands) was installed in each pot. Samplers were inserted diagonally into the tailings profile, with the porous membrane section extending into the lower unamended tailings layer, as shown in Figure 1. Prior to each extraction, pots were watered to approximately 70% of field capacity and allowed to equilibrate for 24 h under greenhouse conditions. TPW was collected annually at the end of each growing season over the seven-year growth period by applying suction to the sampler tubing. A 50 mL TPW sample was collected from each pot.

Immediately after collection, pH and electrical conductivity (EC) were measured on fresh samples using calibrated pH and EC electrodes (Orion 370, Thermo Fisher Scientific, Inc.; VWR 61161-362, respectively). Dissolved organic carbon (DOC) and dissolved nitrogen (DN) were measured on fresh, nonacidified aliquots using a TOC analyzer (Shimadzu TOC-5000A). A 15 mL aliquot of each TPW sample was acidified to 2% with trace-metal-grade nitric acid for elemental analysis. Dissolved Pb, Zn, and Cu concentrations were determined by inductively coupled plasma optical emission spectroscopy (ICP-OES; PerkinElmer Avio 200).

### 2.5 Tailings Core Collection and Analysis after Seven Years

At the end of the seventh growing season, tailings cores were collected from each pot at upper, middle, and lower depth increments. The upper increment corresponded to the amended tailings layer, whereas the middle and lower increments represented unamended tailings layers below the amendment zone. A 1.5 cm diameter soil core sampler was used to collect three cores from each depth increment, as shown in Figure 1. The core sampler was sterilized with 70% ethanol between samples. The three cores from each depth increment were composited, homogenized, and split into subsamples for geochemical, mineralogical, and PLFA analyses. Subsamples for PLFA analysis were stored frozen at −80 °C until extraction, whereas subsamples for the other analyses were air-dried, ground, and sieved before analysis. The depth-specific sampling approach was used to distinguish direct amendment effects in the upper layer from indirect downward effects associated with root development, pore-water transport, and organic carbon deposition.

#### 2.5.1 Organic Carbon and PLFA Analysis

Organic carbon was determined on tailings subsamples using the dichromate wet oxidation Walkley–Black method.^41^ This method was selected because the high carbonate content of the tailings complicated organic carbon determination by high-temperature combustion, while Walkley–Black has been shown to provide reliable organic carbon estimates in calcareous soils.^42^ PLFA analysis was conducted by the University of Missouri’s Soil Health Assessment Center (SHAC) following the high-throughput method of Buyer and Sasser.^43^ Briefly, lipids were extracted using a Bligh–Dyer solvent extraction with an internal standard. Phospholipids were separated from neutral lipids and glycolipids by solid-phase extraction, converted to fatty acid methyl esters, and analyzed by gas chromatography (GC; Agilent 7890A) using the MIDI Sherlock microbial identification system. Individual PLFA peaks were reported as pmol g^–1^ dry sample and converted to nmol g^–1^ where appropriate. Total PLFA was used as an index of viable microbial biomass, and microbial groups and fatty acid types were assigned using the MIDI/SHAC classification scheme. AMF-associated PLFA was represented by 16:1ω5c, fungal PLFA by 18:2ω6,9c, and bacterial groups were estimated from assigned Gram-positive, Gram-negative, and actinobacterial markers. PLFA-derived microbial groups were interpreted as broad community indicators rather than direct taxonomic measurements.

#### 2.5.2 Total Metal and Sequential Extraction Analysis

Aqua-regia-extractable metal concentrations were determined by digesting 1 g of tailings with 28 mL of aqua regia (3:1 HCl:HNO_3_) for 2 h at 120 °C. Metal fractionation was determined using a modified Tessier sequential extraction procedure.^44–46^ Briefly, 1 g of air-dried tailings was sequentially extracted with reagents targeting five fractions: F1, exchangeable; F2, carbonate-associated; F3, Fe/Mn oxide-associated; F4, organic/sulfide-associated; and F5, aqua-regia-extractable residual/nonsilicate-bound. After each extraction step, samples were centrifuged, and the supernatant was collected, filtered through a 0.45 μm membrane, and acidified for elemental analysis. The remaining solid residue was rinsed, centrifuged again, and the rinse solution was discarded before proceeding to the next extraction step. Detailed extractants and extraction conditions are provided in Table S1. Aqua-regia digests and sequential extracts were analyzed for Pb, Zn, Cu, Fe, and Mn by triple quadrupole inductively coupled plasma mass spectrometry (ICP-MS; Agilent 8900 ICP-QQQ).

#### 2.5.3 X-ray Diffraction (XRD)

Tailings samples from the upper amended layer and the middle and lower unamended layers were analyzed by powder X-ray diffraction (XRD; PANalytical X’Pert Pro). Air-dried, finely ground samples were homogenized and front-loaded into standard powder sample holders. XRD patterns were collected using Cu Kα radiation (λ = 1.5418 Å) in θ–θ configuration, with the XRD tube operated at 40 kV and 45 mA. Patterns were collected from 5° to 70° 2θ with a step size of 0.02° 2θ and an approximate counting time of 0.5–1 s per step. Mineral phases were identified by comparing measured diffraction peaks with reference patterns. XRD was used qualitatively to provide mineralogical context for carbonate buffering and sequential extraction results, rather than to assign Pb, Zn, or Cu to specific mineral hosts.

### 2.6 Analytical Quality Assurance and Quality Control

Analytical quality assurance and quality control procedures included procedural blanks, duplicate and spiked samples, and calibration verification standards during elemental analyses. Limits of detection (LOD) and quantification (LOQ) for ICP-OES were, in μg L^–1^, 5.5 and 16.8 for Pb, 2.5 and 7.6 for Zn, and 1.9 and 5.7 for Cu, respectively. Corresponding ICP-MS LOD and LOQ values were, in μg L^–1^, 0.04 and 0.12 for Pb, 0.13 and 0.40 for Zn, 0.28 and 0.86 for Cu, 0.34 and 1.04 for Fe, and 0.13 and 0.39 for Mn, respectively. Accuracy of the aqua-regia digestion was evaluated using the certified reference material ERM-CC136a (Sewage Sludge–Metals), with mean recoveries of 101.7% for Pb, 96.6% for Zn, 105.9% for Cu, 95.0% for Fe, and 96.2% for Mn. Sequential-extraction mass-balance recoveries, calculated as the summed concentration of F1–F5 relative to the corresponding independently measured aqua-regia-extractable concentration (Table S2), averaged 93.0 ± 7.7% for Pb, 90.3 ± 8.2% for Zn, 91.2 ± 7.9% for Cu, 96.8 ± 9.5% for Fe, and 98.4 ± 10.3% for Mn. Additional QA/QC procedures are provided in the Supporting Information.

### 2.7 Statistical Analysis

Statistical analyses were conducted in R (version 4.6.1).^47^ Linear mixed-effects models were fitted using the nlme package^48^ and estimated marginal means with pairwise comparisons were obtained using the emmeans package.^49^

Repeated pore-water measurements collected over seven years were analyzed using linear mixed-effects models^50^ with treatment, year, and the treatment × year interaction as fixed effects and pot identity as a random effect. Candidate within-pot correlation and residual variance structures were evaluated using the Akaike information criterion (AIC)^51^ and likelihood ratio tests. The final models used an independent correlation structure for pH; an autoregressive first-order [AR(1)] correlation structure for Pb and Zn; and an AR(1) correlation structure with treatment-specific residual variances for EC, DOC, DN, and Cu.

Endpoint variables measured at a single sampling time at year 7, including organic carbon, PLFA-based microbial indicators, sequential extraction fractions, and total metal concentrations, were analyzed separately within each tailings layer. Variables meeting parametric assumptions, after transformation when necessary, were analyzed using one-way ANOVA followed by Tukey-adjusted pairwise comparisons. Variables that did not meet parametric assumptions after transformation were analyzed using Kruskal–Wallis tests followed by Dunn’s multiple comparisons.

Model assumptions were evaluated using residual diagnostics appropriate to each analysis, including Q–Q plots, residual-versus-fitted plots, Shapiro–Wilk tests for residual normality, and variance homogeneity assessments when appropriate. Transformations were applied when necessary, with statistical inference conducted on the transformed scale and descriptive statistics reported on the original scale. Effect sizes were calculated as partial eta-squared (η^2^p) for fixed effects in mixed-effects models, eta-squared (η^2^) for ANOVA,^52^ and epsilon-squared (ε^2^) for Kruskal–Wallis analyses.^53^ Statistical significance was determined at *p* < 0.05. Detailed statistical outputs and effect size estimates are provided in Tables S3–S6 in the Supporting Information.

## 3 Results and Discussion

### 3.1 Temporal Effects of Treatments and Revegetation on Tailings Pore-Water Chemistry

The untreated tailings and amendments differed strongly in properties relevant to pore-water chemistry (Table 1). The tailings were circumneutral to slightly alkaline, low in organic matter and cation exchange capacity, and enriched in Pb, Zn, and Cu, whereas biosolids supplied soluble nutrients, salts, organic matter, and metals, and biochar contributed alkaline, carbon-rich, sorptive material. These contrasts were reflected in the seven-year TPW responses (Figures 2 and 3). Linear mixed-effects models showed significant treatment and year effects for pH, EC, DOC, and DN (all *p* ≤ 0.0035). Treatment × year interactions were significant for EC, DOC, and DN (*p* ≤ 0.010), but not for pH (*p* = 0.417) (Table S3).

**Figure 2:**
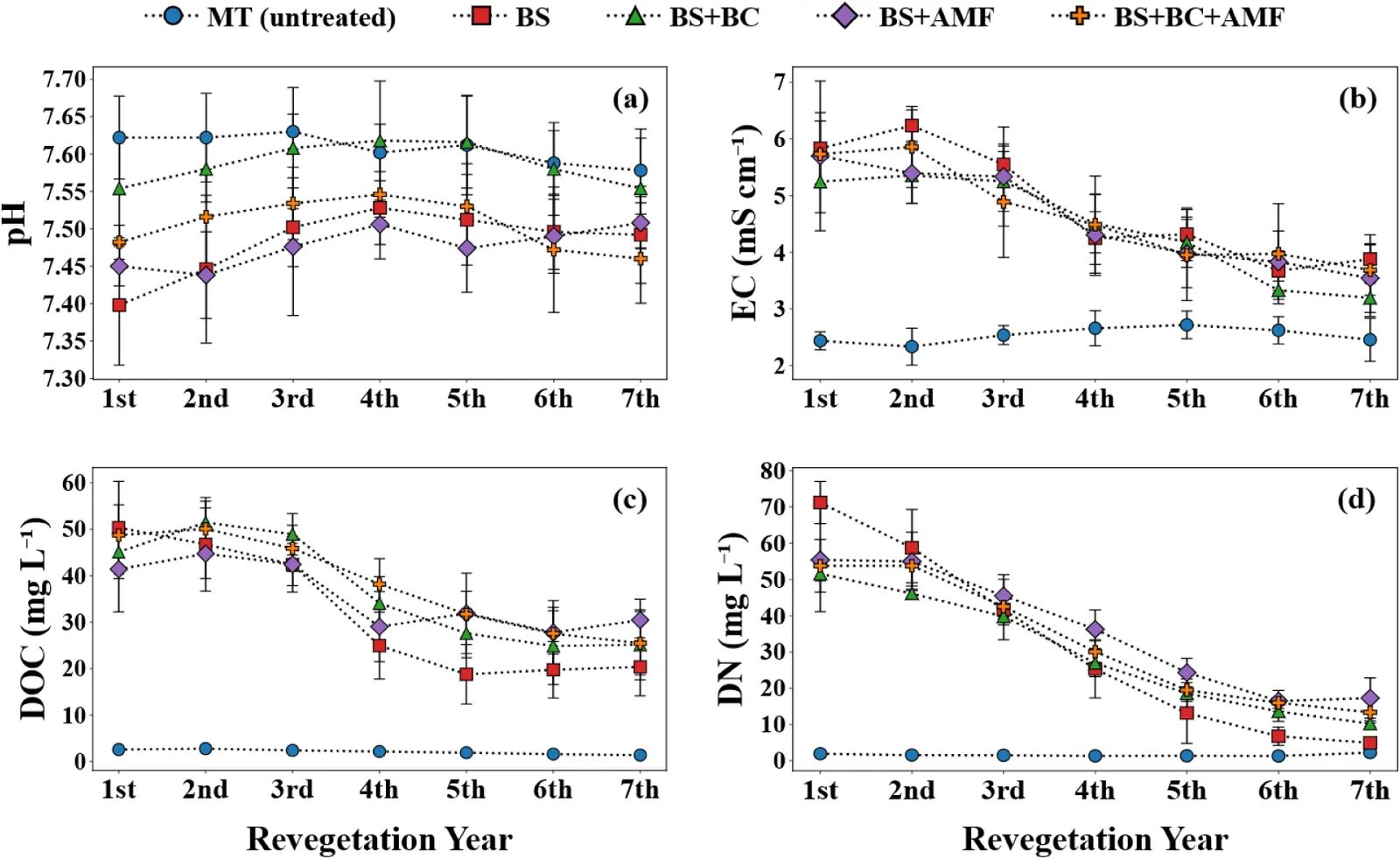
Temporal changes in tailings pore-water (a) pH, (b) electrical conductivity (EC), (c) dissolved organic carbon (DOC), and (d) dissolved nitrogen (DN) over seven years of amendment-assisted revegetation. Points represent treatment means (*n* = 5) ± SD. Mixed-effects model results and Tukey-adjusted post hoc treatment comparisons are reported in Tables S3 and S4, respectively.

**Figure 3:**
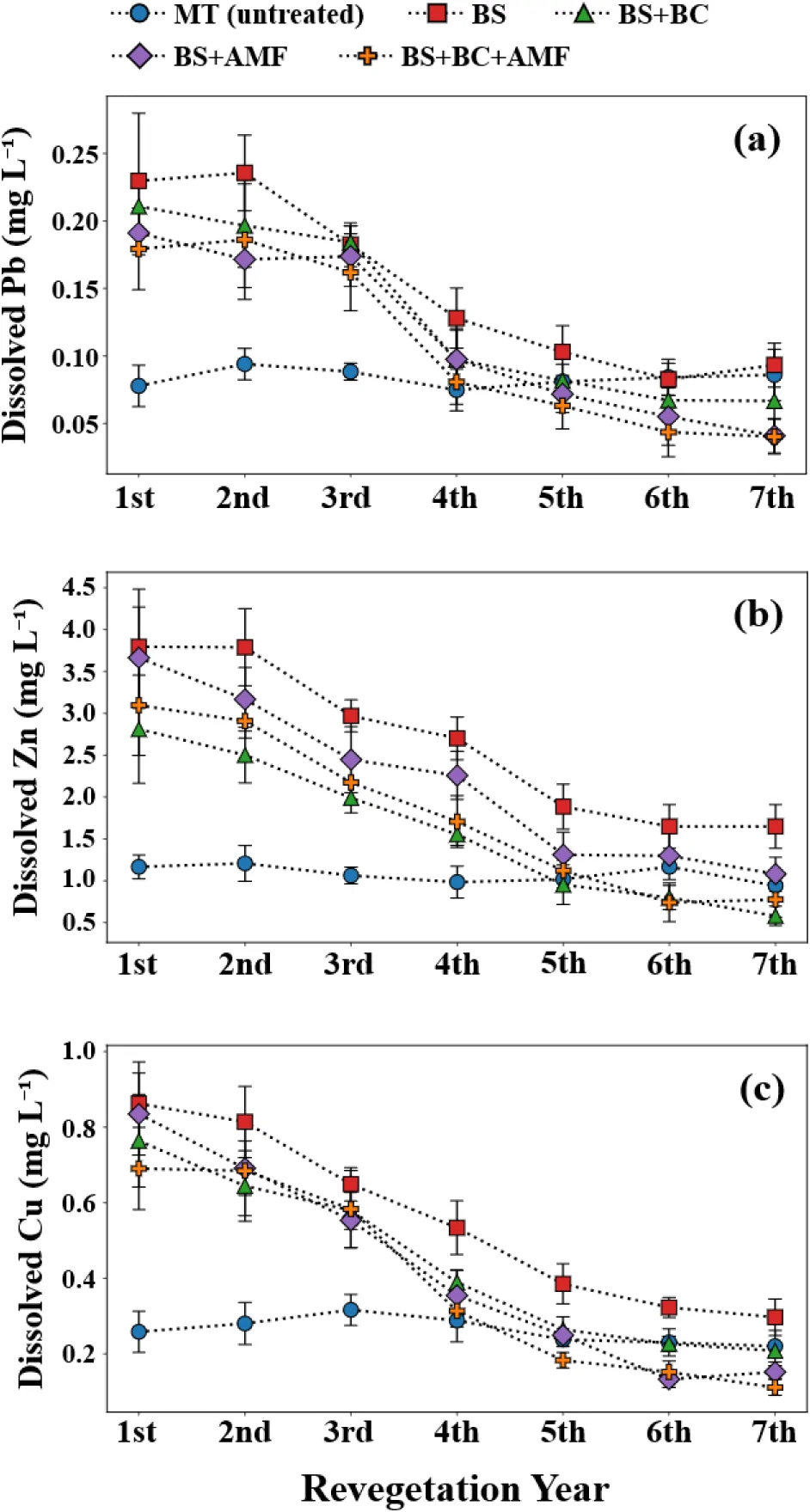
Temporal changes in tailings pore-water dissolved concentrations of (a) Pb, (b) Zn, and (c) Cu over seven years of amendment-assisted revegetation. Points represent treatment means (*n* = 5) ± SD. Mixed-effects model results and Tukey-adjusted post hoc treatment comparisons are reported in Tables S3 and S4, respectively.

TPW pH remained narrowly circumneutral to slightly alkaline, generally around 7.4–7.6. MT and BS+BC had the highest overall pH, whereas BS, BS+AMF, and BS+BC+AMF had lower values (Table S4). This modest pH depression is consistent with the lower pH of the biosolids amendment and may also reflect organic acid production during decomposition and rhizosphere development.^16,54^ However, persistent circumneutral pH is consistent with strong carbonate buffering in the dolomite-rich tailings matrix.^55^

EC, DOC, and DN showed a clear amendment-associated pulse. During the first three years, amended-treatment means were 4.89–6.24 mS cm^–1^ for EC, 41.4–51.5 mg L^–1^ for DOC, and 39.8–71.2 mg L^–1^ for DN, compared with MT means of 2.33–2.54 mS cm^–1^, 2.39–2.77 mg L^–1^, and 1.49–1.93 mg L^–1^, respectively. By year seven, amended-treatment means declined to 3.20–3.88 mS cm^–1^ for EC, 20.37–30.46 mg L^–1^ for DOC, and 4.93–17.27 mg L^–1^ for DN. These trends are consistent with an initially salt-, nutrient-, and DOC-rich TPW environment following amendment application, followed by a decline in readily soluble constituents over time that may reflect processes such as depletion, retention, redistribution, and biological assimilation.

The early elevation of soluble constituents is consistent with the dual role of biosolids as a fertility amendment and a source of labile nutrients, salts, DOC, and metals.^16,17^ However, the later TPW patterns suggest that DOC dynamics were not controlled solely by depletion of biosolids-derived soluble organic matter. Although DOC declined substantially from early year values, DOC remained relatively higher in some combined-amendment treatments than in BS alone by the end of the experiment, consistent with continued contributions from vegetation and microbial processing. Organic carbon amendments and vegetation establishment may influence dissolved organic matter composition, microbial associations, mineral–organic interactions, and aggregation during biogeochemical development in the residuals.^56–58^ Thus, the subsequent decline in EC, DOC, and DN may reflect a transition from an initially amendment-dominated soluble phase toward a TPW environment increasingly influenced by biological and mineralogical processes, rather than simple loss of amendment-derived constituents.^59^

### 3.2 Dissolved Pb, Zn, and Cu Decreased over Time with Combined Amendment Treatments

Linear mixed-effects models showed significant treatment and year effects for dissolved Pb, Zn, and Cu (*p* < 0.0001), with significant treatment × year interactions for all three metals (*p* < 0.0001; Table S3), indicating that treatment differences varied over time. Dissolved Pb, Zn, and Cu followed the same broad temporal pattern as EC and DOC: amended treatments generally showed higher dissolved metals during the first two to three years, followed by substantial attenuation (Figure 3). The early increase was most evident in BS, consistent with its high salinity, DOC, metal content, and slightly lower pH, and with previous biosolids-assisted phytostabilization studies showing that biosolids can improve plant growth while temporarily increasing DOC and dissolved metals.^16,17^ During the first three years, amended-treatment means ranged from 0.162 to 0.236 mg L^–1^ for Pb, 1.99 to 3.80 mg L^–1^ for Zn, and 0.553 to 0.863 mg L^–1^ for Cu, compared with lower MT means of 0.078–0.094, 1.06–1.21, and 0.258–0.316 mg L^–1^, respectively.

This early mobilization did not persist. By year seven, dissolved metals were generally lowest in treatments containing BC and/or AMF. Dissolved Pb decreased to 0.040–0.067 mg L^–1^ in BS+BC, BS+AMF, and BS+BC+AMF; concentrations were significantly lower in BS+AMF and BS+BC+AMF than in MT and BS, while BS+BC showed an intermediate response (Figure 3 and Table S4). Dissolved Zn declined to 0.581–1.081 mg L^–1^ in the combined treatments, with concentrations significantly lower in BS+BC and BS+BC+ AMF than in BS, while BS+AMF showed an intermediate response. Dissolved Cu showed the clearest attenuation, declining to 0.111–0.208 mg L^–1^ in the combined treatments compared with 0.297 mg L^–1^ in BS; concentrations were significantly lower in BS+BC, BS+AMF, and BS+BC+AMF than in BS, with the lowest concentration in BS+BC+AMF. Thus, amendment-assisted revegetation produced a transient mobilization phase followed by substantial attenuation of dissolved metals, particularly when biosolids were combined with BC and/or AMF.

The later-stage decline in dissolved metals despite sustained DOC in some combined treatments suggests that DOC concentration alone did not explain the observed metal patterns. The divergence between DOC and dissolved-metal patterns may reflect changes in the relative importance of dissolved organic constituents, sorption, mineral association, and biological processes as the tailings developed.^56,57,59^ The persistence of higher dissolved Zn and Cu in BS relative to treatments containing BC and/or AMF further suggests that biosolids alone provided less effective long-term metal attenuation.

The metal-specific patterns are consistent with the contrasting geochemical behavior of Pb, Zn, and Cu. Pb is generally less mobile under circumneutral to alkaline conditions because of strong sorption and carbonate association.^60^ Zn is generally more mobile than Pb under circumneutral to alkaline conditions and may remain partly exchangeable or carbonate-associated, depending on pH, carbonate buffering, and weathering state.^4,60^ Cu can be strongly complexed by organic ligands and may also respond to DOM-driven changes in metal release and fractionation.^57^ These differences provide context for the greater persistence of dissolved Zn and Cu in BS relative to treatments containing BC and/or AMF. Biochar may complement biosolids by increasing sorptive capacity and promoting metal retention through mechanisms such as adsorption, ion exchange, surface complexation, and related retention processes.^61–63^ In AMF-containing treatments, the lower dissolved-metal concentrations are consistent with mycorrhizosphere-associated stabilization mechanisms reported in the literature, including effects on root development, metal retention, and soil aggregation.^21,24,64,65^ AMF-containing treatments were generally associated with greater root development throughout the tailings profile, including increased root proliferation and deeper penetration into unamended layers, as qualitatively illustrated by representative opened-pot images (Figure S1). Together with the higher OC and AMF-associated PLFA observed in AMF-containing treatments after seven years (Figures 4 and S2), these responses are consistent with a broader biologically influenced zone and may have contributed to longer-term biological and geochemical attenuation. Overall, the year-seven dissolved-metal patterns indicate a shift from amendment-associated mobilization toward greater metal attenuation in treatments containing BC and/or AMF.

**Figure 4:**
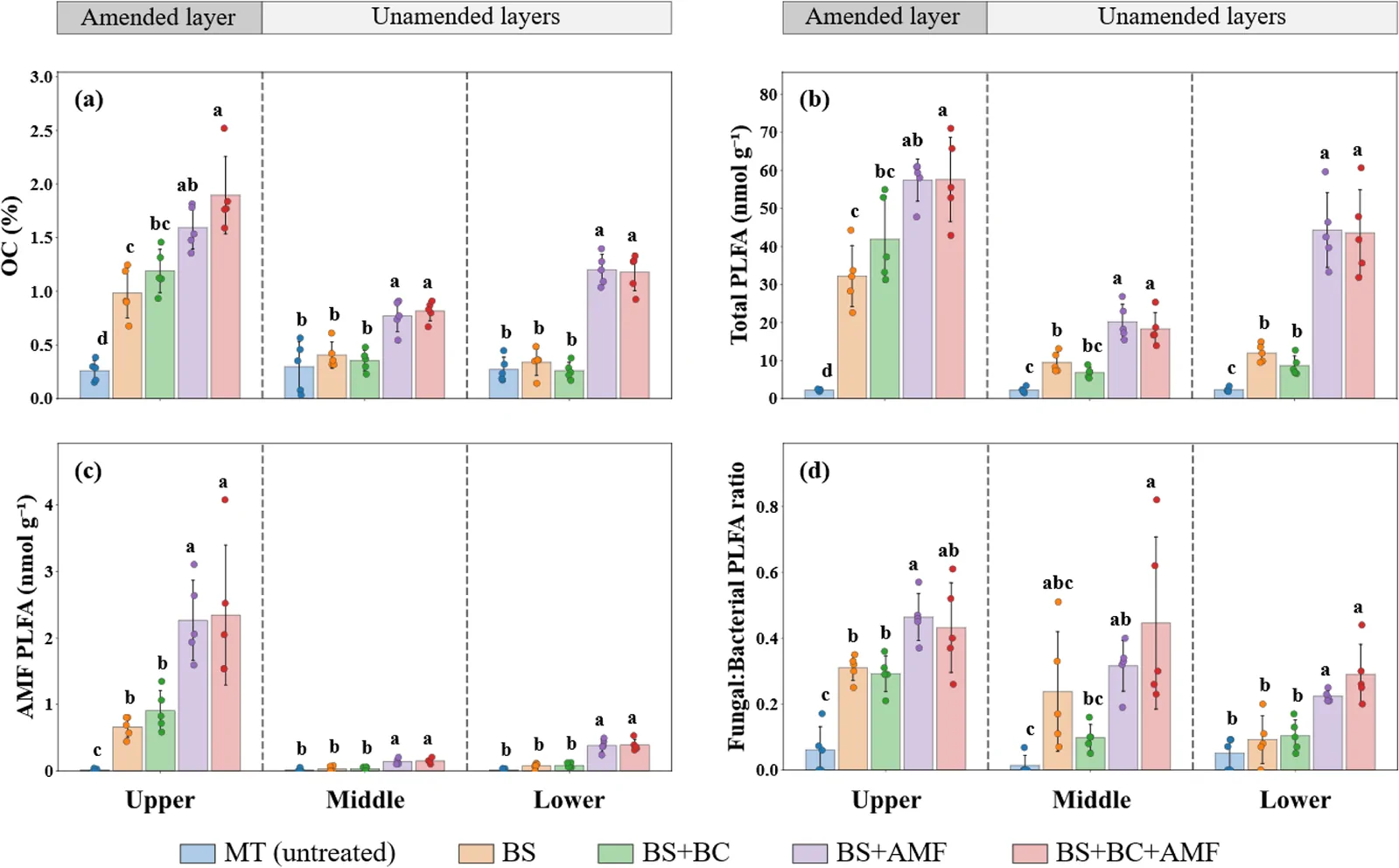
Organic carbon and microbial community indicators after seven years of amendment-assisted revegetation across tailings layers: (a) organic carbon (OC), (b) total PLFA, (c) AMF-associated PLFA, and (d) fungal:bacterial PLFA ratio. Bars represent treatment means (*n* = 5) ± SD; points show individual replicates. Different letters indicate significant differences among treatments within each tailings layer based on Tukey’s HSD post hoc test following one-way ANOVA (*p* < 0.05).

### 3.3 Organic Carbon and Microbial Indicators Showed Depth-Dependent Tailings Development

After seven years, amendment-assisted revegetation was associated with increased OC and microbial biomass indicators relative to untreated tailings (Figures 4 and S2). Treatment effects were significant for OC, total PLFA, AMF-associated PLFA, and fungal:bacterial PLFA ratio in all three tailings layers (*p* ≤ 0.001; Table S5). Compared with unamended tailings, amended treatments supported sustained plant establishment after seven years, as illustrated qualitatively in Figure S3. MT remained low in OC across all depths, with mean values near 0.3%. In the upper amended layer, OC ranged from 0.98 to 1.90% across amended treatments, with the highest values in AMF-containing treatments. OC enrichment also extended into the middle and lower unamended layers, particularly in AMF-containing treatments. In the lower layer, OC reached 1.18–1.20% in AMF-containing treatments, compared with ≤0.34% in MT and non-AMF amended treatments. Because amendments were incorporated only into the upper layer, the deeper OC enrichment is unlikely to result from direct amendment mixing alone. The pattern is consistent with belowground C transfer associated with root growth, root turnover, rhizodeposition, microbial processing, and possibly downward movement of dissolved or particulate organic constituents.^66^ Opened-pot observations after seven years indicated that roots extended below the 8 cm amended layer into the underlying unamended tailings, with deeper root penetration qualitatively evident in AMF-containing treatments (Figure S1). Mycorrhizal hyphae can extend the biologically influenced zone beyond roots and contribute to C stabilization through hyphal entanglement, altered root architecture, fungal products, hyphal turnover, and associated effects on soil structure.^64,67,68^ Recent tailings studies similarly show that plant colonization, microbial processing, and AMF activity can promote organic matter stabilization and organo-mineral association during tailings development.^59,65,69^

PLFA analysis, a biochemical indicator of viable microbial biomass and community structure, showed a strong treatment response.^70^ Total PLFA in MT remained low across depths, near 2.3 nmol g^–1^. In the upper layer, total PLFA increased to 32.20–41.92 nmol g^–1^ in non-AMF amended treatments and to 57.43–57.61 nmol g^–1^ in AMF-containing treatments. In the lower unamended layer, total PLFA reached 43.56–44.30 nmol g^–1^ in AMF-containing treatments, compared with ≤ 11.97 nmol g^–1^ in MT and non-AMF amended treatments. Thus, microbial biomass indicators were elevated beyond the directly amended surface layer, including in the unamended deeper tailings. AMF-associated PLFA was nearly absent in MT but increased strongly in inoculated treatments, reaching 2.27–2.35 nmol g^–1^ in the upper layer and remaining detectable in the lower layer. Representative microscopy images of AMF-associated structures within roots provide qualitative evidence of AMF colonization in AMF-inoculated treatments (Figure S4). Together with the observed deeper root penetration, these responses are consistent with AMF-associated biological influence across the tailings profile, although its spatial extent cannot be resolved from the present observations. Fungal:bacterial PLFA ratios followed a similar pattern, with the highest or among the highest values generally observed in AMF-containing treatments. AMF-containing treatments generally showed higher Gram-positive, Gram-negative, actinomycete, and fungal PLFA in the middle and lower unamended layers. These groups provide broad functional indicators rather than species-level identification. Gram-negative bacteria are commonly associated with rhizosphere activity and utilization of more labile C sources, whereas Gram-positive bacteria and actinomycetes can contribute to decomposition and turnover of more complex organic substrates.^43,70^ The higher fungal and AMF-associated PLFA responses are consistent with greater fungal involvement in belowground C and nutrient cycling and may have contributed to organic matter stabilization through C inputs and mechanisms such as aggregate-associated protection.^68,71^

Together, the OC, PLFA, root observations, and AMF-associated measurements support the interpretation that amendment-assisted revegetation promoted biological development beyond the amended surface layer. The stronger responses in AMF-containing treatments are consistent with a contribution from root- and mycorrhiza-associated processes and provide a plausible pathway for longer-term organic matter accumulation and microbial establishment at depth.

### 3.4 Metal Stabilization through Reduced Lability and Repartitioning after Seven Years of Revegetation

Because aqua-regia-extractable Pb, Zn, and Cu remained elevated after seven years (Table S2), treatment effectiveness is best interpreted as stabilization rather than removal. Evidence for stabilization comes from the combined decline in TPW metal concentrations, reduced readily and potentially labile pools, and repartitioning among sequential extraction fractions.

The concentrations of the readily labile F1 (exchangeable) and potentially labile F1+F2 (exchangeable + carbonate-associated) pools clarify the treatment and layer responses (Figure S5). Layer-specific analyses showed significant treatment effects for F1 and F1+F2 for all three metals in all three layers (*p* ≤ 0.0029; Table S6). Across treatments and depths, F1 was much smaller than F1+F2, indicating that the carbonate-associated F2 pool accounted for most of the potentially labile fraction. For Pb, amended treatments generally showed lower F1 and F1+F2 concentrations than untreated tailings, with differences also observed in the middle and lower unamended layers. For Zn, F1+F2 remained more persistent across treatments and depths, indicating a greater contribution of the carbonate-associated pool. For Cu, exchangeable concentrations were negligible or absent, and treatment effects were expressed mainly through differences in potentially labile and less labile pools.

The full sequential extraction profiles further indicate that Pb stabilization was associated with both reduced exchangeability and greater contributions from less labile pools (Figure 5). Pb was dominated by carbonate-associated and Fe/Mn oxide-associated fractions, with smaller organic/sulfide-associated and residual/nonsilicate-bound contributions. In amended treatments, Pb generally showed a lower contribution from the exchangeable pool and greater contributions from Fe/Mn oxide-associated and residual/nonsilicate-bound fractions. Although organic/sulfide-associated Pb remained a minor pool, its slight increase in amended or biologically developed unamended layers coincided with higher OC, microbial indicators, and qualitative observations of deeper root penetration in AMF-inoculated treatments (Figure S1). Overall, the Pb fractionation patterns are consistent with retention predominantly within carbonate- and oxide-associated operational pools, with a smaller contribution from organic/sulfide-associated pools. This interpretation is consistent with prior Pb–Zn tailings studies showing Pb retention in carbonate, oxide-associated, residual, or PbS-like forms depending on mineralogical context and weathering conditions.^4,60^

**Figure 5:**
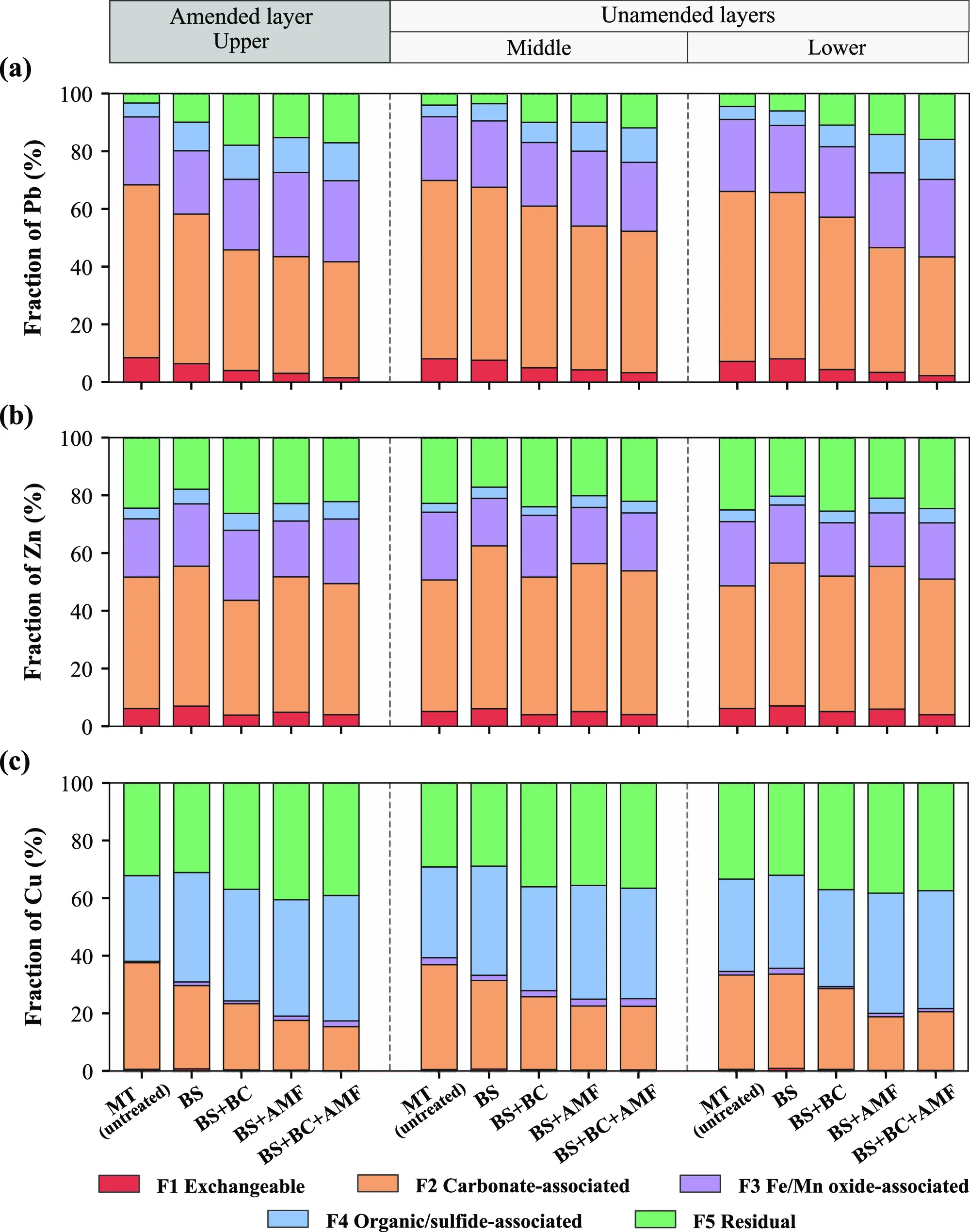
Relative distribution of (a) Pb, (b) Zn, and (c) Cu after seven years of amendment-assisted revegetation. Bars show the mean percentage contribution of each sequential extraction fraction to the summed F1–F5 pool for each treatment and tailings layer. Fractions are operationally defined as F1, exchangeable; F2, carbonate-associated; F3, Fe/Mn oxide-associated; F4, organic/sulfide-associated; and F5, residual.

Zn showed a more persistent potentially labile pool than Pb. Although exchangeable Zn remained relatively small, F1+F2 persisted across treatments and depths, indicating a greater contribution of the carbonate-associated pool than for Pb or Cu. However, the lower potentially labile Zn in combined treatments containing BC and/or AMF, together with the lower dissolved Zn observed in TPW during later sampling years, suggests that biosolids alone improved fertility but did not provide the same long-term Zn attenuation as treatments combining nutrient addition with sorptive and biological functions. This behavior is consistent with previous Pb–Zn tailings studies showing that Zn can be distributed across carbonate, oxide-associated, and residual fractions and may be sensitive to pH, carbonate buffering, and weathering conditions.^5,46^

Cu behaved differently from Pb and Zn. Exchangeable Cu was negligible or absent, and treatment differences were expressed mainly through differences in the relative contributions of carbonate-associated, organic/sulfide-associated, and residual/nonsilicate-bound fractions. This pattern coincided with the OC and PLFA responses observed after revegetation, particularly in AMF-containing treatments and deeper layers, where Cu showed greater representation in organic/sulfide-associated and residual/nonsilicate-bound pools. Together with the TPW data, this pattern is consistent with Cu stabilization through reduced aqueous expression and greater representation in less labile pools rather than changes in exchangeable Cu. This interpretation is consistent with the affinity of Cu for organic matter and reduced sulfur-containing phases and provides geochemical context for the Cu-bearing sulfide mineralogy of the Viburnum Trend.^55,57^

Together, the layer-dependent sequential extraction patterns complement the OC, PLFA, and root observations. Root penetration, rhizosphere development, organic carbon inputs, and pore-water-mediated processes may have contributed to treatment-associated metal differences below the zone of direct amendment incorporation. Overall, the sequential extraction results complement the aqueous and biological findings by showing depth-dependent and metal-specific differences in metal lability and partitioning after seven years of amendment-assisted revegetation. Declining TPW metal concentrations, reduced labile pools in several amended treatments, biological development below the amended layer, and greater representation of metals in oxide-associated, organic/sulfide-associated, and residual/nonsilicate-bound pools are consistent with reduced exposure potential under the experimental conditions. These operational fractions are interpreted as comparative indicators of relative lability and retention rather than direct mineralogical speciation.^44,72,73^

### 3.5 Fe/Mn Fractionation and XRD Provided Geochemical and Mineralogical Context for Metal Retention

Fe and Mn fractionation provided geochemical context for interpreting the distribution of Pb, Zn, and Cu among operationally defined pools (Figures 6a, b and S6). Fe was dominated by the residual/nonsilicate-bound fraction, with a smaller but consistent Fe/Mn oxide-associated pool. This pattern indicates that most Fe occurred in relatively resistant operational pools, whereas the oxide-associated fraction represents a potentially reactive pool that can contribute to trace metal retention through sorption and associated processes.^44,46,72^ The presence of Pb and Zn in the Fe/Mn oxide-associated fraction is consistent with retention by reactive oxide phases, although sequential extraction cannot distinguish specific mineral hosts or reaction mechanisms.^60,72,73^ Mn showed more pronounced differences among treatments than Fe in its operationally defined fraction distribution. In untreated tailings, Mn was mainly associated with the reducible Fe/Mn oxide-associated fraction, whereas amended treatments showed a greater contribution from the residual/nonsilicate-bound pool, particularly in the upper amended layer. Because Mn oxides are redox-sensitive and can influence trace-metal retention, this redistribution is consistent with changes in Mn-associated operational pools under amendment-assisted revegetation.^7^

**Figure 6:**
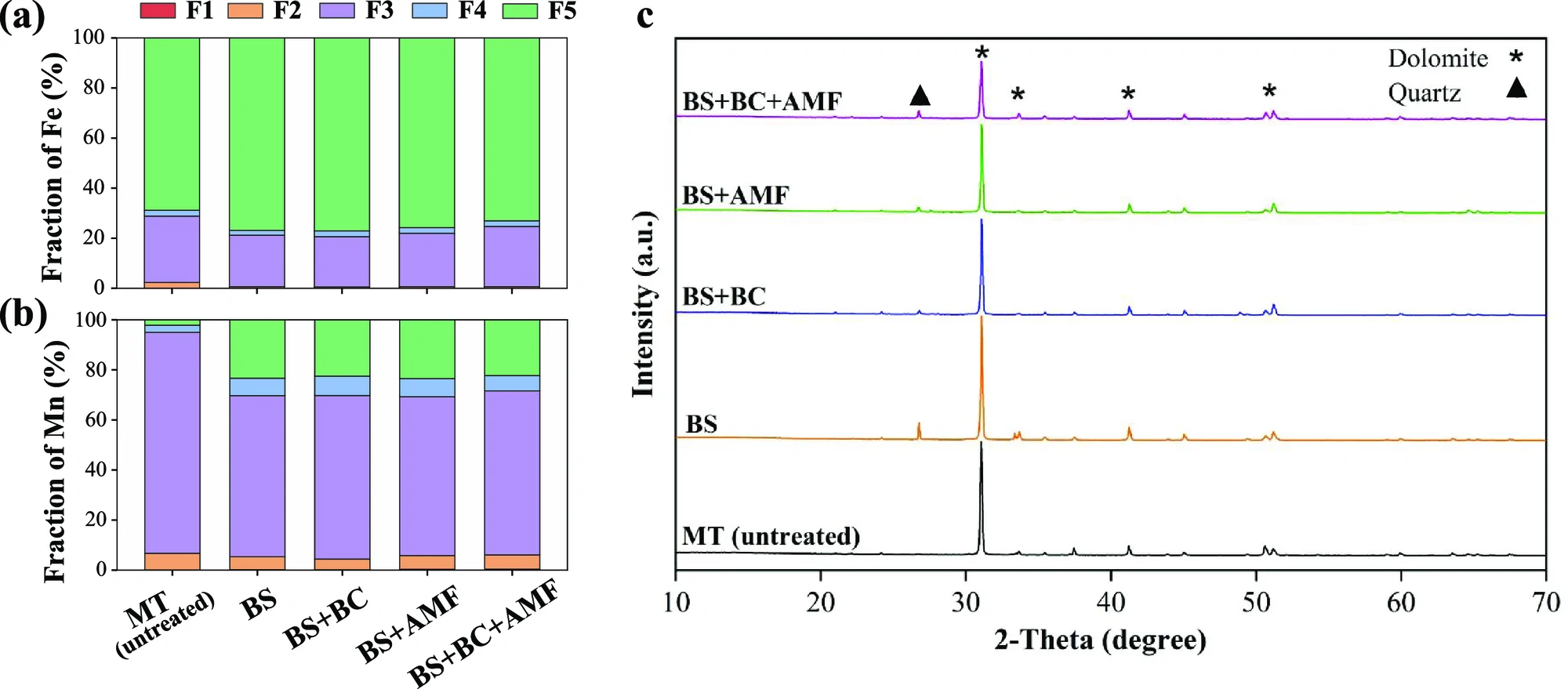
Fe and Mn fractionation and mineralogical context of the upper amended tailings layer after seven years of amendment-assisted revegetation. Stacked bars show the mean percentage distribution of (a) Fe and (b) Mn among sequential extraction fractions. Fractions are operationally defined as F1: exchangeable; F2: carbonate-associated; F3: Fe/Mn oxide-associated; F4: organic/sulfide-associated; and F5: residual. Panel (c) shows representative XRD patterns for each treatment.

XRD patterns from the upper, middle, and lower tailings layers were dominated by dolomite, with quartz also present (Figures 6c and S7). Dolomite and quartz were the only clearly identifiable crystalline phases, showing that the carbonate-rich mineral assemblage remained dominated by dolomite after seven years of amendment-assisted revegetation. The dominance of dolomite is consistent with the carbonate-buffered TPW chemistry and provides mineralogical context for the substantial carbonate-associated Pb and Zn pools observed in the sequential extraction. However, carbonate association should not be interpreted as permanent immobilization because F1+F2 represents a potentially labile pool that may respond to changes in pH, rhizosphere chemistry, ionic strength, or dissolved organic matter conditions.^73^ The XRD patterns did not reveal clear treatment-related changes in the major crystalline phases that could account for the observed metal repartitioning. Minor or poorly crystalline phases, if present, were not clearly identifiable and may have been masked by the dominant dolomite and quartz reflections. The XRD analysis was qualitative and was not intended to quantify mineral abundances or treatment-related changes in mineral abundance. Therefore, XRD is best interpreted as mineralogical context rather than direct evidence of Pb, Zn, or Cu speciation, while the sequential extraction fractions represent operationally defined pools rather than discrete mineralogical species.

### 3.6 Environmental Implications and Study Limitations

The seven-year results show that amendment-assisted revegetation was associated with a transition from early amendment-associated mobilization toward greater biological development and metal attenuation. Although biosolids initially increased EC, DOC, DN, and dissolved metals, these responses attenuated over time, particularly in treatments containing BC and/or AMF. Concurrent increases in OC, PLFA-based microbial indicators, and fungal:bacterial ratios in the middle and lower unamended layers, together with metal-specific repartitioning of Pb, Zn, and Cu, indicate that long-term stabilization was associated with coupled aqueous, solid-phase, and biological changes extending beyond the directly amended surface layer (Figures 2–4 and S2). Successful phytostabilization should therefore be evaluated in terms of both contaminant stabilization and substrate development, because surface vegetation alone may overstate restoration progress if deeper tailings remain carbon-poor, biologically inactive, and geochemically labile. Deeper root penetration, AMF-associated PLFA, microbial proliferation, and changes in pore-water chemistry were consistent with biological and geochemical influence extending into underlying unamended tailings, including carbon accumulation, microbial habitat development, and potential C and nutrient cycling. However, the relative contributions of roots, AMF, microbial processes, and pore-water-mediated transport cannot be resolved from the present data.

From a management perspective, the results support complementary amendment strategies that combine fertility, sorption, biological development, and metal retention. Biosolids supported revegetation, but BS alone maintained higher dissolved Zn and Cu by year seven, whereas treatments containing BC and/or AMF showed stronger long-term metal attenuation and biological development. Restoration performance should therefore consider pore-water chemistry, solid-phase lability, OC accumulation, and microbial indicators in addition to vegetation establishment and total metal concentrations. Because the biosolids also contained elevated Pb, Zn, and Cu, their metal loading should be considered as a potential secondary contamination source under environmental conditions that promote remobilization, such as changes in pH or dissolved organic matter. Field-scale economic feasibility will further depend on the local and sustained availability of amendment materials, transport distances, application rates, and site-preparation requirements, and the cost, availability, and scalability of AMF inoculation; these factors require site-specific evaluation before large-scale implementation.

Field-scale restoration should also consider diverse native plant communities rather than relying on the single species evaluated here. Multispecies communities may provide complementary rooting patterns, carbon inputs, and nutrient-acquisition strategies that influence microbial development, ecosystem development, and succession, consistent with field restoration studies showing benefits of AMF-associated plant–microbe interactions for native plant establishment and community development.^74,75^ N-fixing legumes, such as the purple prairie clover used herein, can contribute to nitrogen availability in nutrient-limited tailings, while AMF-mediated belowground interactions may influence nutrient acquisition and interactions among co-occurring plant species.^76,77^ However, the greenhouse pot design does not reproduce field-scale hydrologic variability, preferential flow, seasonal climatic conditions, or spatial root and plant-community development, and the study did not directly quantify leaching fluxes or field-scale metal transport. Accordingly, these responses represent stabilization under the experimental conditions rather than direct evidence of reduced metal export at field scale. Field studies are needed to determine whether they persist under variable hydrologic and climatic conditions and translate into reduced metal transport and sustained ecosystem development.

## Supplementary Material



## Notes

AMF inoculum composition; sequential extraction solutions and conditions; detailed QA/QC procedures and analytical performance; representative root observations; representative microscopy images of AMF colonization; plant growth; total and labile Pb, Zn, and Cu concentrations; statistical analyses and post hoc comparisons; PLFA-based microbial community indicators; Fe/Mn fractionation; XRD patterns (PDF)
