# Supplementary material for: Long-Term Biogeochemical Stabilization of Carbonate-Bearing Pb–Zn–Cu Mine Tailings by Amendment-Assisted Phytostabilization
Source: Environ Sci Technol. 2026 Oct 15;60(43):30636–48. doi: 10.1021/acs.est.6c08282 (PMC13738792; doi:10.1021/acs.est.6c08282)
Supplement: Supplementary file 1 [file es6c08282_si_001.pdf]

Title: Long-Term Biogeochemical Stabilization of Carbonate-Bearing Pb–Zn–Cu Mine Tailings by Amendment-Assisted Phytostabilization

Mariam Al-Lami[1*], Genevieve Sutton[2], Kwame Awuah-Offei[3], Lana Alagha[3], Joel Burken[1*]

[1]*Department of Civil, Architectural and Environmental Engineering, Missouri University of Science and Technology, Rolla, MO, 65409.*

[2]*The Doe Run Company, St. Louis, MO, 63146*

[3]*Department of Mining & Explosives Engineering, Missouri University of Science & Technology, Rolla, MO, 65409.*

***Corresponding authors:** Mariam Al-Lami; Email: mkan87@mst.edu and Joel Burken; Email: burken@mst.edu

AMF inoculum composition

The AMF inoculum used in this study was obtained from MycoBloom (Lawrence, KS, USA) and contained spores, hyphal mycelium, and colonized root fragments from multiple AMF species, including *Acaulospora trappei*, *Gigaspora gigantea*, *Claroideoglomus claroideum* (formerly *Glomus claroideum*), *Funneliformis mosseae* (formerly *Glomus mosseae*), *Rhizophagus intraradices* (formerly *Glomus intraradices*), *Gigaspora margarita*, and *Glomus mortonii*.

Table S1. Reagents, extraction conditions, and targeted geochemical fractions in the sequential extraction protocol.

**Fraction**	**Extracted pool**	**Extractant and procedure**	**L:S ratio ($mL\ g^{-1}$)**	**Temperature**	**Time**
F1	Exchangeable	1.0 M $MgCl_2$ adjusted to pH 7.0; continuous agitation	20:1	Room temperature	1 h
F2	Carbonate-associated	1.0 M NaOAc adjusted to pH 5.0 with HOAc; continuous agitation with periodic pH adjustment to 5.0	20:1	Room temperature	5 h
F3	Reducible Fe/Mn oxide-associated	0.04 M $NH_2OH \cdot HCl$ in 25% (v/v) HOAc; occasional agitation	20:1	96 °C	5 h
F4	Oxidizable organic/sulfide-associated	Oxidation with 0.02 M HNO_3 and 30% H_2O_2 acidified to pH 2 with HNO_3, followed by extraction with 3.2 M NH_4OAc in 20% (v/v) HNO_3; occasional agitation	36:1*	85 °C	5 h
F5	Aqua-regia-extractable residual/non-silicate-bound	Aqua regia digestion of the residual solid after F4 using 3:1 $HCl:HNO_3$	28:1	120 °C	2 h

Note. The L:S ratio for F4 reflects the combined liquid volume used during oxidation and subsequent ammonium acetate extraction.
After each extraction step, samples were centrifuged, the supernatant was collected for analysis, and the remaining residue was rinsed and centrifuged before the next extraction step.

Quality assurance and quality control

Quality assurance and quality control procedures were followed for pore-water chemistry, aqua-regia digestion, sequential extraction, ICP-OES, ICP-MS, and PLFA analyses. High-purity standards and trace-metal-grade reagents were used for elemental analyses. All glassware, digestion vessels, extraction tubes, and other materials used for sample preparation were soaked overnight in 8% HNO_3 and rinsed thoroughly with Milli-Q water before use. Procedural blanks, duplicate samples, and spiked samples were included with analytical batches, as appropriate. Calibration verification standards were analyzed during TOC, ICP-OES, and ICP-MS runs to monitor instrument performance.

Limits of detection (LOD) and quantification (LOQ) were estimated using the standard-deviation-of-response and calibration-slope approach described by Shrivastava and Gupta.[1] Accuracy of the aqua-regia digestion was evaluated using the certified reference material ERM®-CC136a (Sewage Sludge–Metals).

Sequential-extraction mass balance was evaluated by comparing the summed concentrations recovered in F1–F5 for each sample with the corresponding independently measured aqua-regia-extractable concentration. Recovery ranges were 77.9–112.0% for Pb, 77.7–111.2% for Zn, 78.3–107.2% for Cu, 79.2–115.5% for Fe, and 76.9–118.6% for Mn.

For sequential extraction, samples were centrifuged after each extraction step, the supernatant was collected for analysis, and the remaining solid residue was rinsed and centrifuged before the subsequent extraction step to minimize carryover between fractions. For PLFA analysis, quality-control procedures at the University of Missouri Soil Health Assessment Center included procedural blanks, soil reference standards, internal-standard recovery checks, and continuing gas chromatographic calibration verification using the MIDI Sherlock system. These procedures were used to monitor contamination, extraction consistency, instrument performance, and analytical precision.

(a) (b)

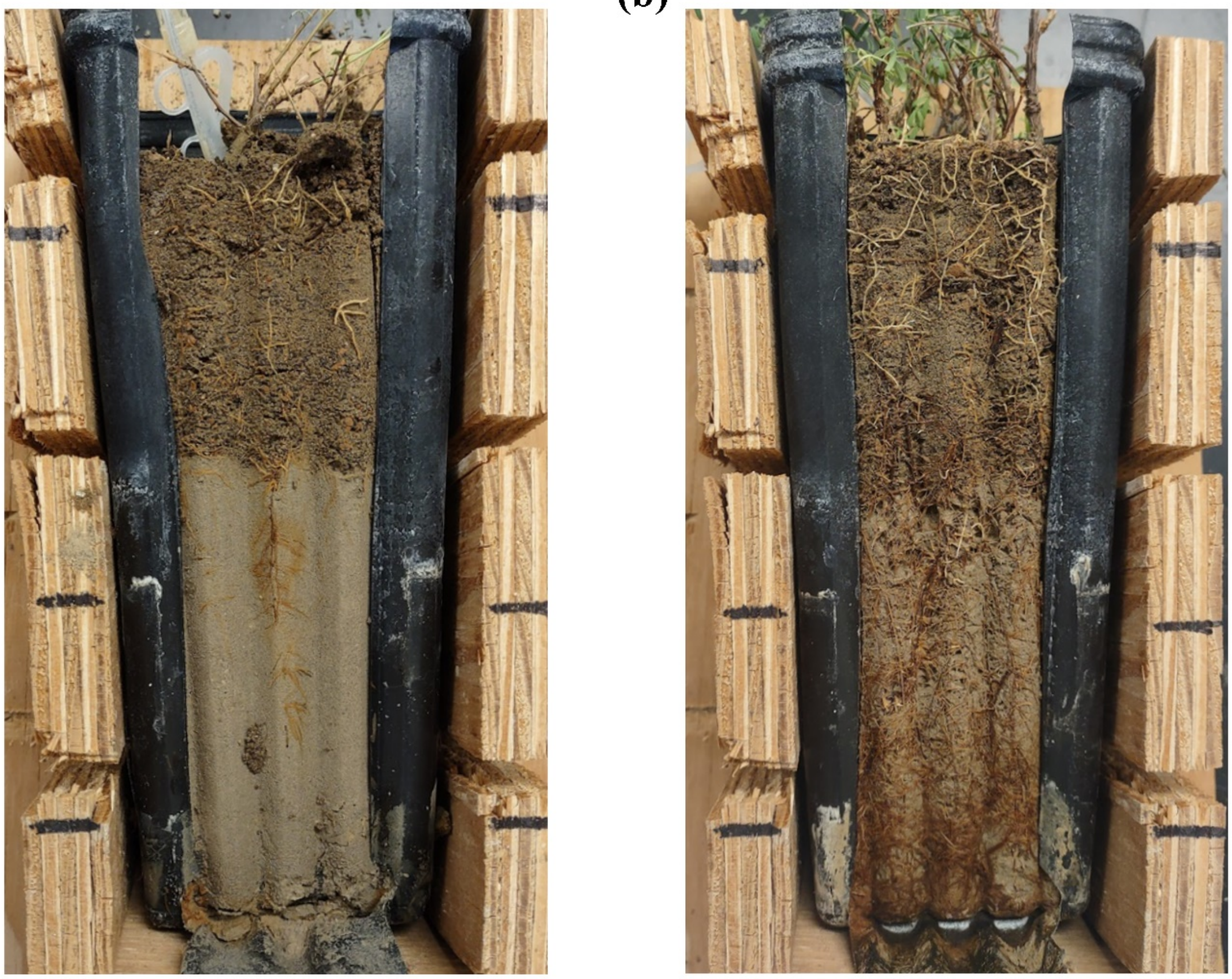

Figure S1. Representative opened-pot images of root architecture after seven years of growth in tailings: **(a)** BS without AMF and **(b)** BS with AMF. The images are representative qualitative observations and were not used for quantitative measurements of root biomass, root length, or AMF colonization. They provide qualitative visual evidence of greater root proliferation and deeper root penetration in the AMF-containing treatment, consistent with greater belowground development and an expanded root-influenced zone extending into unamended tailings layers.

Table S2. Total Pb, Zn, and Cu concentrations in tailings after seven years of amendment-assisted revegetation. Values are mean ± SD.

Treatment	Layer	Aqua regia-extractable metal concentration (mg kg^{-1})		
		**Pb**	**Zn**	**Cu**
**MT (untreated)**	Upper	4250 ± 493	1011 ± 70	572 ± 65
	Middle	4503 ± 344	993 ± 74	565 ± 133
	Lower	4162 ± 186	879 ± 116	530 ± 92
**BS**	Upper	3945 ± 380	1171 ± 139	728 ± 76
	Middle	3946 ± 390	1108 ± 44	571 ± 35
	Lower	4354 ± 520	1084 ± 119	519 ± 134
**BS+BC**	Upper	3554 ± 483	1146 ± 109	603 ± 94
	Middle	4007 ± 461	1080 ± 73	483 ± 130
	Lower	4147 ± 142	1028 ± 97	666 ± 57
**BS+AMF**	Upper	3849 ± 255	1100 ± 67	753 ± 156
	Middle	4046 ± 333	1033 ± 72	635 ± 66
	Lower	4178 ± 216	990 ± 67	595 ± 38
**BS+BC+AMF**	Upper	3312 ± 582	1110 ± 116	699 ± 62
	Middle	3964 ± 284	957 ± 38	579 ± 75
	Lower	4152 ± 786	996 ± 117	566 ± 65

Table S4. Post hoc treatment comparisons based on linear mixed-effects models of tailings pore-water parameters during the seven-year monitoring period.

Parameter	Year	MT (untreated)	BS	BS+BC	BS+AMF	BS+BC+AMF
**pH**	Overall	7.608 ± 0.013 **a**	7.482 ± 0.013 **b**	7.587 ± 0.013 **a**	7.477 ± 0.013 **b**	7.506 ± 0.013 **b**
**EC**	Y1	2.44 ± 0.16 **b**	5.83 ± 0.63 **a**	5.25 ± 0.55 **a**	5.70 ± 1.32 **a**	5.73 ± 0.59 **a**
	Y2	2.33 ± 0.33 **b**	6.24 ± 0.28 **a**	5.36 ± 0.49 **a**	5.40 ± 0.53 **a**	5.86 ± 0.72 **a**
	Y3	2.54 ± 0.17 **b**	5.55 ± 0.36 **a**	5.25 ± 0.53 **a**	5.34 ± 0.88 **a**	4.89 ± 0.98 **a**
	Y4	2.66 ± 0.31 **b**	4.25 ± 0.47 **a**	4.51 ± 0.52 **a**	4.31 ± 0.71 **a**	4.49 ± 0.86 **a**
	Y5	2.72 ± 0.24 **b**	4.32 ± 0.46 **a**	4.18 ± 0.44 **a**	3.98 ± 0.60 **a**	3.95 ± 0.80 **a**

Table S3. Fixed-effect tests from linear mixed-effects models of tailings pore-water parameters during the seven-year monitoring period.

Parameter	Fixed effect	*df*	*F*-value	*p*-value	η_p^2
**pH**	Treatment	4, 20	23.49	<0.0001	0.824
	Year	6, 144	3.42	0.0035	0.125
	Treatment × Year	24, 120	1.04	0.4170	0.173
**EC**	Treatment	4, 20	94.01	<0.0001	0.949
	Year	6, 120	10.35	<0.0001	0.341
	Treatment × Year	24, 120	7.93	<0.0001	0.613
**DOC**	Treatment	4, 20	678.22	<0.0001	0.993
	Year	6, 120	40.09	<0.0001	0.667
	Treatment × Year	24, 120	1.95	0.0100	0.281
**DN**	Treatment	4, 20	1057.96	<0.0001	0.995
	Year	6, 120	60.90	<0.0001	0.753
	Treatment × Year	24, 120	40.00	<0.0001	0.889
**Pb**	Treatment	4, 20	19.78	<0.0001	0.798
	Year	6, 120	102.73	<0.0001	0.837
	Treatment × Year	24, 120	7.20	<0.0001	0.590
**Zn**	Treatment	4, 20	61.87	<0.0001	0.925
	Year	6, 120	150.87	<0.0001	0.883
	Treatment × Year	24, 120	10.06	<0.0001	0.668
**Cu**	Treatment	4, 20	35.05	<0.0001	0.875
	Year	6, 120	392.72	<0.0001	0.952
	Treatment × Year	24, 120	15.37	<0.0001	0.754

Note. η_p^2, partial eta-squared. Treatment, year, and Treatment × Year were evaluated as fixed effects in the linear mixed-effects models. For pH, the nonsignificant Treatment × Year interaction was evaluated using the full interaction model, whereas the reported main effects of Treatment and Year were obtained from the reduced additive model. Statistical significance was evaluated at $p < 0.05$. Model specifications, transformations, covariance structures, residual diagnostics, and software are described in the Statistical Analysis section of the main text.

	Y6	2.62 ± 0.24 **b**	3.67 ± 0.35 **a**	3.33 ± 0.16 ab	3.83 ± 0.55 **a**	3.97 ± 0.88 **a**
	Y7	2.46 ± 0.38 **b**	3.88 ± 0.43 **a**	3.20 ± 0.33 **a**	3.54 ± 0.61 **a**	3.69 ± 0.45 **a**
**DOC**	Y1	2.58 ± 0.39 **b**	50.35 ± 9.98 **a**	45.11 ± 5.72 **a**	41.41 ± 9.20 **a**	48.74 ± 6.55 **a**
	Y2	2.77 ± 0.59 **b**	46.76 ± 10.10 **a**	51.48 ± 4.54 **a**	44.75 ± 5.34 **a**	50.10 ± 4.48 **a**
	Y3	2.39 ± 0.35 **b**	42.40 ± 4.51 **a**	48.94 ± 4.45 **a**	42.46 ± 5.98 **a**	45.90 ± 4.98 **a**
	Y4	2.15 ± 0.28 **c**	24.95 ± 7.18 **b**	34.08 ± 5.73 **ab**	29.02 ± 7.54 **ab**	38.19 ± 5.48 **a**
	Y5	1.89 ± 0.28 **c**	18.79 ± 6.41 **b**	27.62 ± 5.28 **a**	31.89 ± 8.65 **a**	31.75 ± 4.90 **a**
	Y6	1.60 ± 0.15 **b**	19.75 ± 6.08 **a**	24.89 ± 8.31 **a**	27.79 ± 4.66 **a**	27.55 ± 7.08 **a**
	Y7	1.38 ± 0.33 **c**	20.37 ± 6.24 **b**	25.13 ± 7.54 **ab**	30.46 ± 4.49 **a**	25.46 ± 6.82 **ab**
**DN**	Y1	1.93 ± 0.39 **b**	71.20 ± 5.82 **a**	51.58 ± 1.99 **a**	55.39 ± 14.30 **a**	53.76 ± 7.27 **a**
	Y2	1.56 ± 0.19 **b**	58.74 ± 10.60 **a**	46.13 ± 1.17 **a**	54.97 ± 8.08 **a**	53.76 ± 4.70 **a**
	Y3	1.49 ± 0.10 **b**	41.68 ± 8.33 **a**	39.80 ± 2.34 **a**	45.47 ± 5.84 **a**	42.53 ± 3.41 **a**
	Y4	1.33 ± 0.16 **b**	25.31 ± 7.99 **a**	26.94 ± 3.78 **a**	36.25 ± 5.34 **a**	30.06 ± 2.99 **a**
	Y5	1.35 ± 0.15 **c**	13.14 ± 8.38 **b**	18.57 ± 4.81 **a**	24.33 ± 3.91 **a**	19.47 ± 3.09 **a**
	Y6	1.31 ± 0.28 **c**	6.75 ± 2.51 **b**	13.61 ± 2.77 **a**	16.46 ± 2.89 **a**	15.96 ± 1.55 **a**
	Y7	2.23 ± 0.43 **d**	4.93 ± 1.48 **c**	10.21 ± 0.76 **b**	17.27 ± 5.55 **a**	13.36 ± 2.97 **ab**
**Pb**	Y1	0.078 ± 0.015 **b**	0.230 ± 0.050 **a**	0.211 ± 0.020 **a**	0.191 ± 0.016 **a**	0.179 ± 0.030 **a**
	Y2	0.094 ± 0.012 **c**	0.235 ± 0.028 **a**	0.197 ± 0.031 **ab**	0.172 ± 0.021 **b**	0.186 ± 0.044 **ab**
	Y3	0.088 ± 0.006 **b**	0.182 ± 0.016 **a**	0.184 ± 0.012 **a**	0.174 ± 0.022 **a**	0.162 ± 0.028 **a**
	Y4	0.075 ± 0.015 **b**	0.128 ± 0.022 **a**	0.097 ± 0.022 ab	0.097 ± 0.023 ab	0.081 ± 0.017 **b**
	Y5	0.081 ± 0.002 ab	0.103 ± 0.019 **a**	0.082 ± 0.012 ab	0.072 ± 0.014 ab	0.063 ± 0.017 **b**
	Y6	0.084 ± 0.013 **a**	0.083 ± 0.012 **a**	0.067 ± 0.016 ab	0.055 ± 0.021 ab	0.044 ± 0.018 **b**
	Y7	0.086 ± 0.019 **a**	0.093 ± 0.016 **a**	0.067 ± 0.021 ab	0.041 ± 0.013 **b**	0.040 ± 0.013 **b**
**Zn**	Y1	1.17 ± 0.14 **b**	3.80 ± 0.69 **a**	2.81 ± 0.65 **a**	3.66 ± 0.60 **a**	3.10 ± 0.60 **a**
	Y2	1.21 ± 0.21 **c**	3.79 ± 0.46 **a**	2.50 ± 0.33 **b**	3.17 ± 0.38 **ab**	2.91 ± 0.21 **ab**
	Y3	1.06 ± 0.10 **c**	2.97 ± 0.19 **a**	1.99 ± 0.18 **b**	2.45 ± 0.40 **ab**	2.17 ± 0.24 **b**
	Y4	0.98 ± 0.19 **d**	2.70 ± 0.26 **a**	1.55 ± 0.13 **c**	2.26 ± 0.29 **ab**	1.71 ± 0.31 **bc**
	Y5	1.02 ± 0.11 **bc**	1.89 ± 0.27 **a**	0.95 ± 0.23 **c**	1.31 ± 0.27 **b**	1.12 ± 0.13 **bc**
	Y6	1.17 ± 0.02 **b**	1.65 ± 0.26 **a**	0.80 ± 0.14 **c**	1.30 ± 0.28 **ab**	0.74 ± 0.23 **c**
	Y7	0.94 ± 0.14 **bc**	1.65 ± 0.26 **a**	0.58 ± 0.12 **d**	1.08 ± 0.20 **b**	0.78 ± 0.21 **cd**

Table S4. (continued)

Parameter	Year	MT (untreated)	BS	BS+BC	BS+AMF	BS+BC+AMF
**Cu**	Y1	0.258 ± 0.054 **b**	0.863 ± 0.110 **a**	0.764 ± 0.123 **a**	0.835 ± 0.108 **a**	0.691 ± 0.109 **a**
	Y2	0.280 ± 0.056 **b**	0.814 ± 0.094 **a**	0.644 ± 0.093 **a**	0.691 ± 0.072 **a**	0.685 ± 0.119 **a**
	Y3	0.316 ± 0.041 **b**	0.649 ± 0.044 **a**	0.580 ± 0.050 **a**	0.553 ± 0.074 **a**	0.583 ± 0.102 **a**

Table S5. Statistical summary of layer-specific treatment effects on organic carbon and PLFA-based microbial indicators after seven years of amendment-assisted revegetation.

**Response variable**	**Layer**	**Test**	***df***	**Test statistic**	***p*-value**	**Effect size**

Table S4. (continued)

**Parameter**	**Year**	**MT (untreated)**	**BS**	**BS+BC**	**BS+AMF**	**BS+BC+AMF**
	Y4	0.288 ± 0.056 **c**	0.534 ± 0.071 **a**	0.389 ± 0.032 **b**	0.355 ± 0.067 **bc**	0.313 ± 0.042 **bc**
	Y5	0.238 ± 0.019 **bc**	0.385 ± 0.053 **a**	0.265 ± 0.033 **b**	0.249 ± 0.017 **b**	0.183 ± 0.021 **c**
	Y6	0.230 ± 0.036 **b**	0.323 ± 0.027 **a**	0.227 ± 0.020 **b**	0.133 ± 0.022 **c**	0.152 ± 0.029 **c**
	Y7	0.220 ± 0.042 **b**	0.297 ± 0.048 **a**	0.208 ± 0.013 **b**	0.152 ± 0.017 **c**	0.111 ± 0.021 **d**

Note. For EC, DOC, DN, Pb, Zn, and Cu, values are arithmetic means ± SD (n = 5) on the original scale; letters indicate treatment groupings from Tukey-adjusted comparisons of model-derived estimated marginal means among treatments within each year ($p < 0.05$), and groups sharing a letter are not significantly different. Bold letters identify treatment means involved in at least one significant pairwise comparison within that year. Because Treatment × Year was not significant for pH, pH values are reported as overall estimated marginal means ± SE across years with Tukey groupings from the reduced additive model. Transformations, where used, affected inference only; descriptive values are shown on the original scale.

	Upper	ANOVA	4, 20	35.85	<0.001	0.878
**Organic carbon (OC)**	Middle	ANOVA	4, 20	13.84	<0.001	0.735
	Lower	ANOVA	4, 20	71.63	<0.001	0.935
	Upper	ANOVA	4, 20	38.14	<0.001	0.884
**Total PLFA**	Middle	ANOVA	4, 20	29.32	<0.001	0.854
	Lower	ANOVA	4, 20	140.21	<0.001	0.966
	Upper	ANOVA	4, 20	46.40	<0.001	0.903
**AMF-associated PLFA**	Middle	ANOVA	4, 20	17.14	<0.001	0.774
	Lower	ANOVA	4, 20	41.70	<0.001	0.893
	Upper	ANOVA	4, 20	19.22	<0.001	0.794
**Fungal:bacterial PLFA ratio**	Middle	ANOVA	4, 20	6.73	0.001	0.574
	Lower	ANOVA	4, 20	13.72	<0.001	0.733
	Upper	Kruskal–Wallis	4	18.30	0.0011	0.715
**Fungal PLFA**	Middle	Kruskal–Wallis	4	19.80	<0.001	0.790
	Lower	Kruskal–Wallis	4	19.24	<0.001	0.762
	Upper	ANOVA	4, 20	31.22	<0.001	0.862
**Gram-negative PLFA**	Middle	ANOVA	4, 20	49.65	<0.001	0.909
	Lower	ANOVA	4, 20	87.30	<0.001	0.946
	Upper	ANOVA	4, 20	22.15	<0.001	0.816
**Gram-positive PLFA**	Middle	ANOVA	4, 20	19.54	<0.001	0.796
	Lower	ANOVA	4, 20	63.51	<0.001	0.927
	Upper	ANOVA	4, 20	105.61	<0.001	0.955
**Actinomycete PLFA**	Middle	ANOVA	4, 20	53.38	<0.001	0.914
	Lower	ANOVA	4, 20	27.49	<0.001	0.846

Table S5. (continued)

Response variable	Layer	Test	*df*	Test statistic	*p*-value	Effect size

Table S6. Statistical summary of layer-specific treatment effects on total and labile Pb, Zn, and Cu pools after seven years of amendment-assisted revegetation.

**Predator:prey PLFA ratio**	Upper	Kruskal–Wallis	4	20.62	<0.001	0.831
	Middle	Kruskal–Wallis	4	16.59	0.0023	0.630
	Lower	Kruskal–Wallis	4	19.36	<0.001	0.768
**Gram-positive:Gram-negative PLFA ratio**	Upper	ANOVA	4, 20	44.68	<0.001	0.899
	Middle	ANOVA	4, 20	6.43	0.0017	0.563
	Lower	ANOVA	4, 20	23.28	<0.001	0.823
**Saturated:unsaturated PLFA ratio**	Upper	ANOVA	4, 20	68.77	<0.001	0.932
	Middle	ANOVA	4, 20	12.01	<0.001	0.706
	Lower	ANOVA	4, 20	7.86	<0.001	0.611
**Gram-negative bacterial stress index**	Upper	ANOVA	4, 20	6.60	0.0015	0.569
	Middle	ANOVA	4, 20	4.50	0.0093	0.474
	Lower	ANOVA	4, 20	3.71	0.021	0.426

Note. Treatment effects were evaluated separately within each tailings layer. Test statistic values are F values for one-way ANOVA and H values for Kruskal-Wallis tests. Effect sizes are eta-squared (η^2) for ANOVA and epsilon-squared (ε^2) for Kruskal-Wallis tests. Statistical significance was evaluated at $p < 0.05$. Analytical procedures, transformations, and post hoc procedures are described in the Statistical Analysis section of the main text.

Response variable	Layer	Test	*df*	Test statistic	*p*-value	Effect size
**Total Pb**	Upper	ANOVA	4, 20	3.19	0.035	0.390
	Middle	Kruskal–Wallis	4	6.01	0.198	0.101
	Lower	ANOVA	4, 20	0.19	0.938	0.037
**Pb F1**	Upper	ANOVA	4, 20	77.26	<0.001	0.939
	Middle	ANOVA	4, 20	22.46	<0.001	0.818
	Lower	ANOVA	4, 20	28.93	<0.001	0.853
**Pb F1+F2**	Upper	ANOVA	4, 20	15.94	<0.001	0.761
	Middle	ANOVA	4, 20	8.46	<0.001	0.628
	Lower	ANOVA	4, 20	11.49	<0.001	0.697
**Total Zn**	Upper	ANOVA	4, 20	1.73	0.184	0.257
	Middle	ANOVA	4, 20	4.90	0.0064	0.495
	Lower	ANOVA	4, 20	2.54	0.072	0.337
**Zn F1**	Upper	ANOVA	4, 20	53.18	<0.001	0.914
	Middle	ANOVA	4, 20	35.51	<0.001	0.877
	Lower	ANOVA	4, 20	28.49	<0.001	0.851
**Zn F1+F2**	Upper	ANOVA	4, 20	6.52	0.0016	0.566
	Middle	ANOVA	4, 20	7.07	0.0010	0.586
	Lower	ANOVA	4, 20	5.81	0.0029	0.537
**Total Cu**	Upper	ANOVA	4, 20	3.34	0.030	0.401
	Middle	ANOVA	4, 20	1.61	0.212	0.243
	Lower	ANOVA	4, 20	2.47	0.078	0.330
**Cu F1**	Upper	ANOVA	4, 20	71.43	<0.001	0.935
	Middle	ANOVA	4, 20	12.81	<0.001	0.719
	Lower	ANOVA	4, 20	61.35	<0.001	0.925
**Cu F1+F2**	Upper	ANOVA	4, 20	17.59	<0.001	0.779
	Middle	ANOVA	4, 20	5.80	0.0029	0.537
	Lower	ANOVA	4, 20	14.85	<0.001	0.748

Note. Treatment effects were evaluated separately within each tailings layer. Total metal concentrations were determined by aqua regia extraction; F1 represents the exchangeable fraction and F1+F2 the exchangeable plus carbonate-associated pool. Test statistic values are *F* values for one-way ANOVA and *H* values for Kruskal–Wallis tests. Effect sizes are eta-squared (η^2) for ANOVA and epsilon-squared (ε^2) for Kruskal-Wallis tests. Statistical significance was evaluated at $p < 0.05$. Analytical procedures, transformations, and post hoc procedures are described in the Statistical Analysis section of the main text.

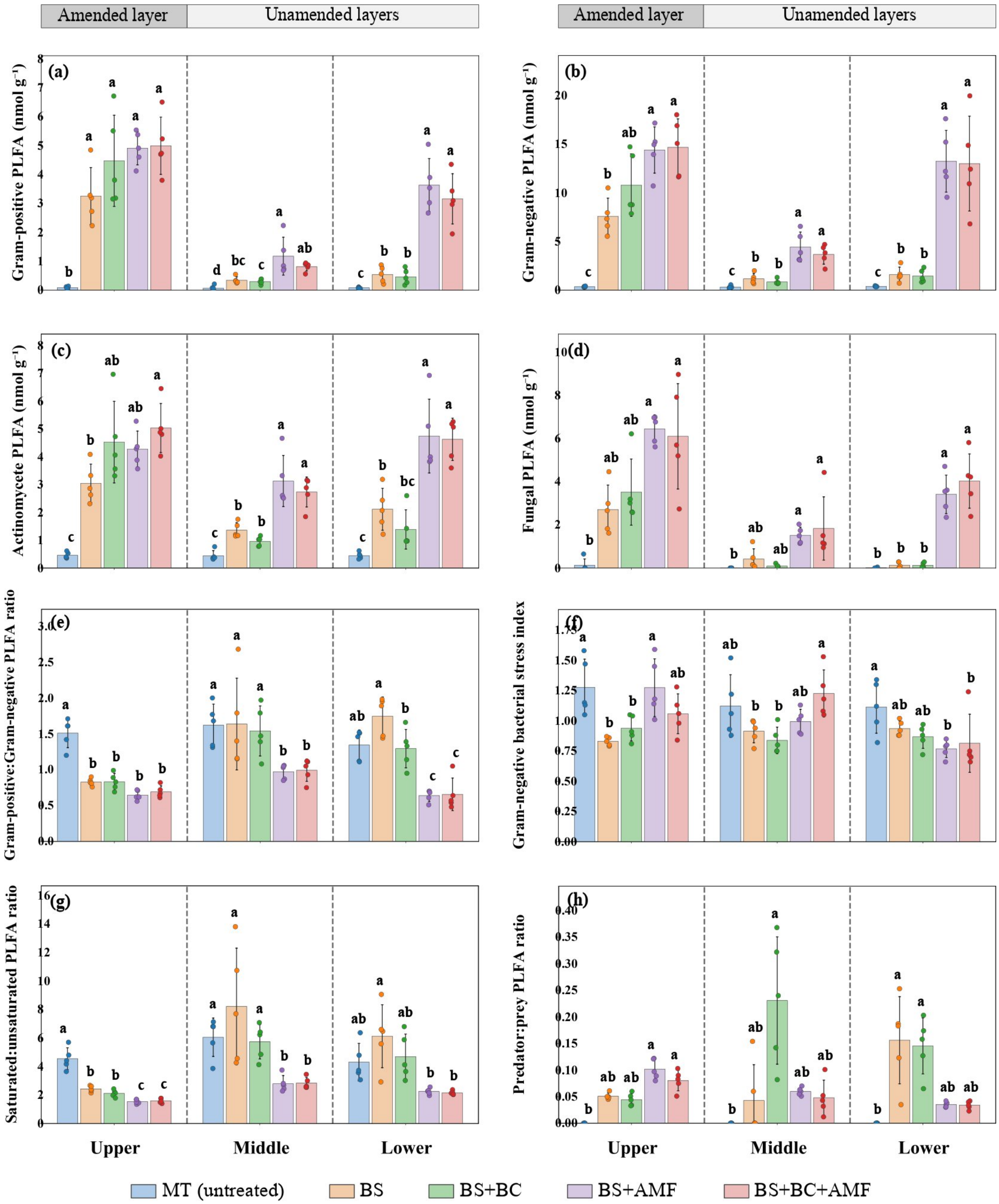

Figure S2. Microbial community indicators after seven years of amendment-assisted revegetation across tailings layers. Bars show treatment means (n = 5) and error bars indicate standard deviations for **(a)** Gram-positive PLFA, **(b)** Gram-negative PLFA, **(c)** actinomycete PLFA, **(d)** fungal PLFA, **(e)** Gram-positive:Gram-negative PLFA ratio, **(f)** Gram-negative bacterial stress index, **(g)** saturated:unsaturated PLFA ratio, and **(h)** predator:prey PLFA ratio in

the upper amended layer and middle and lower unamended tailings layers. Points represent individual replicates. Different letters above the bars indicate significant differences among treatments within each layer based on one-way ANOVA or Kruskal–Wallis tests, as appropriate ($p < 0.05$).

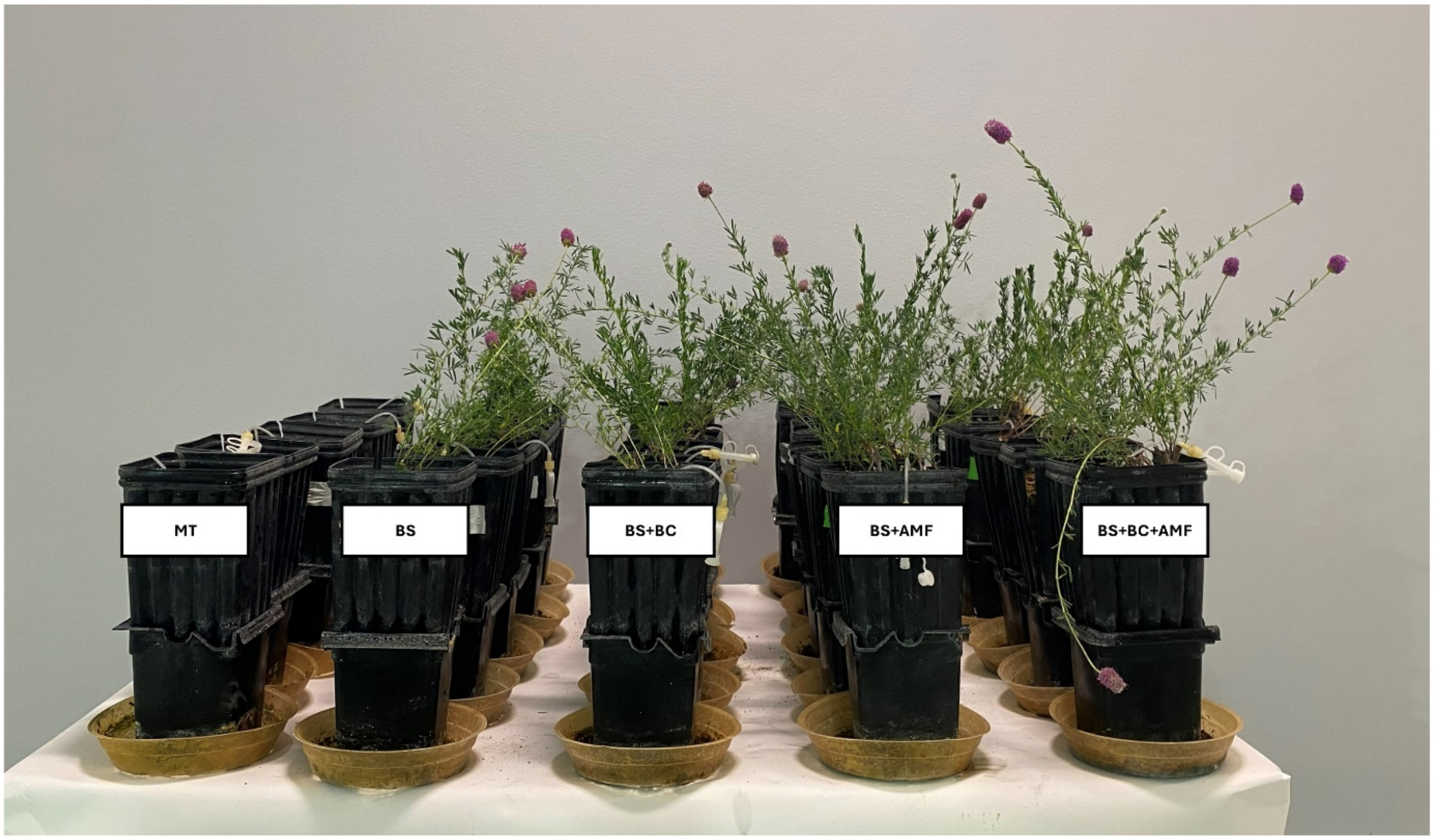

Figure S3. Greenhouse pot images showing purple prairie clover (*Dalea purpurea*) growth after seven years in untreated tailings (MT) and amendment-assisted revegetation treatments: biosolids (BS), biosolids + biochar (BS+BC), biosolids + arbuscular mycorrhizal fungi (BS+AMF), and biosolids + biochar + arbuscular mycorrhizal fungi (BS+BC+AMF). The images provide representative qualitative visual evidence of sustained plant establishment and growth among treatments.

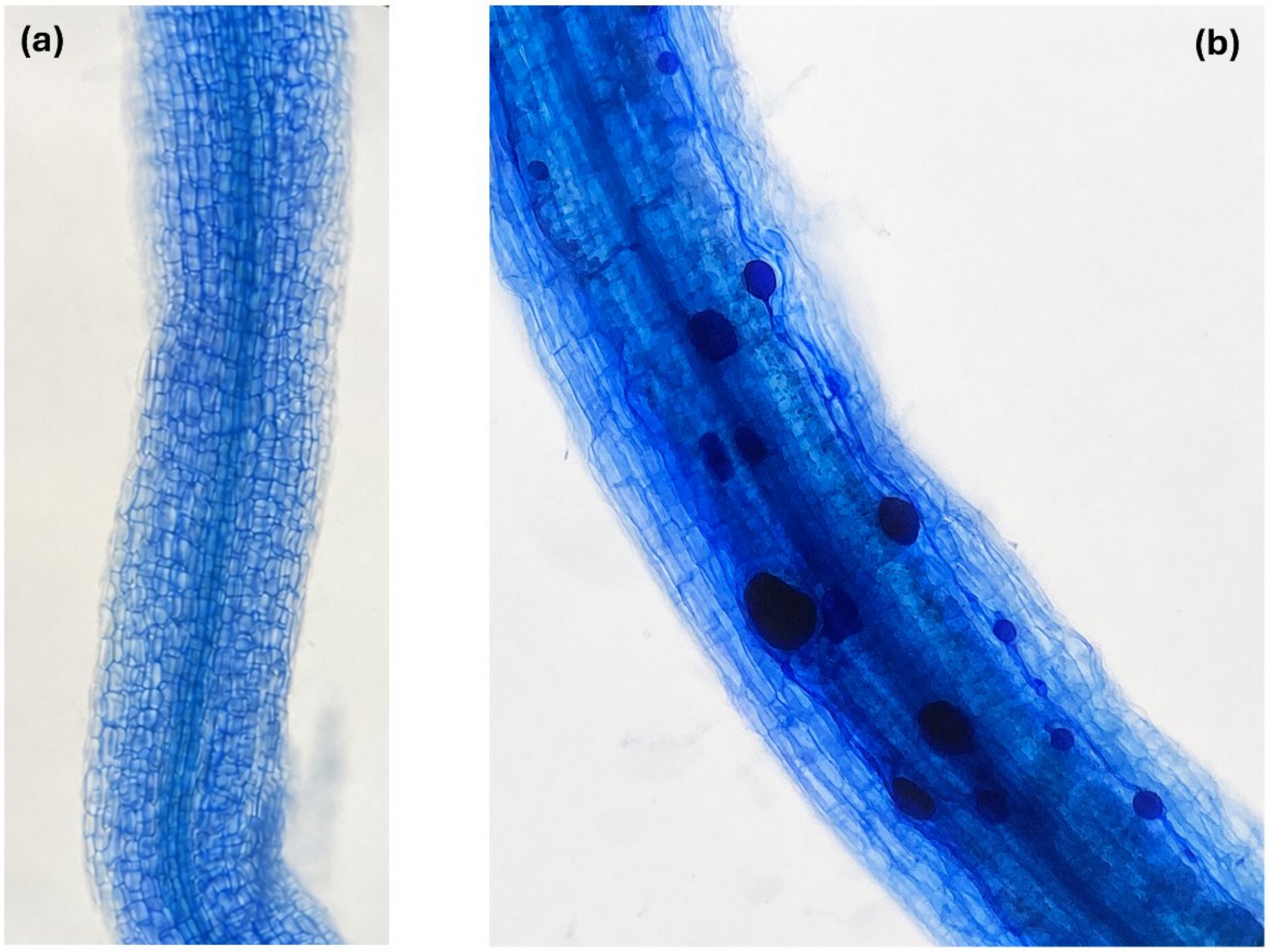

Figure S4. Trypan blue-stained root segments showing representative **(a)** nonmycorrhizal root of *Dalea purpurea* plant growing in non-inoculated treated tailings and **(b)** mycorrhizal colonized root of *Dalea purpurea* plant growing in AMF-inoculated treated tailings. The images provide qualitative evidence of AMF colonization and were not used for quantitative or layer-resolved assessment of colonization.

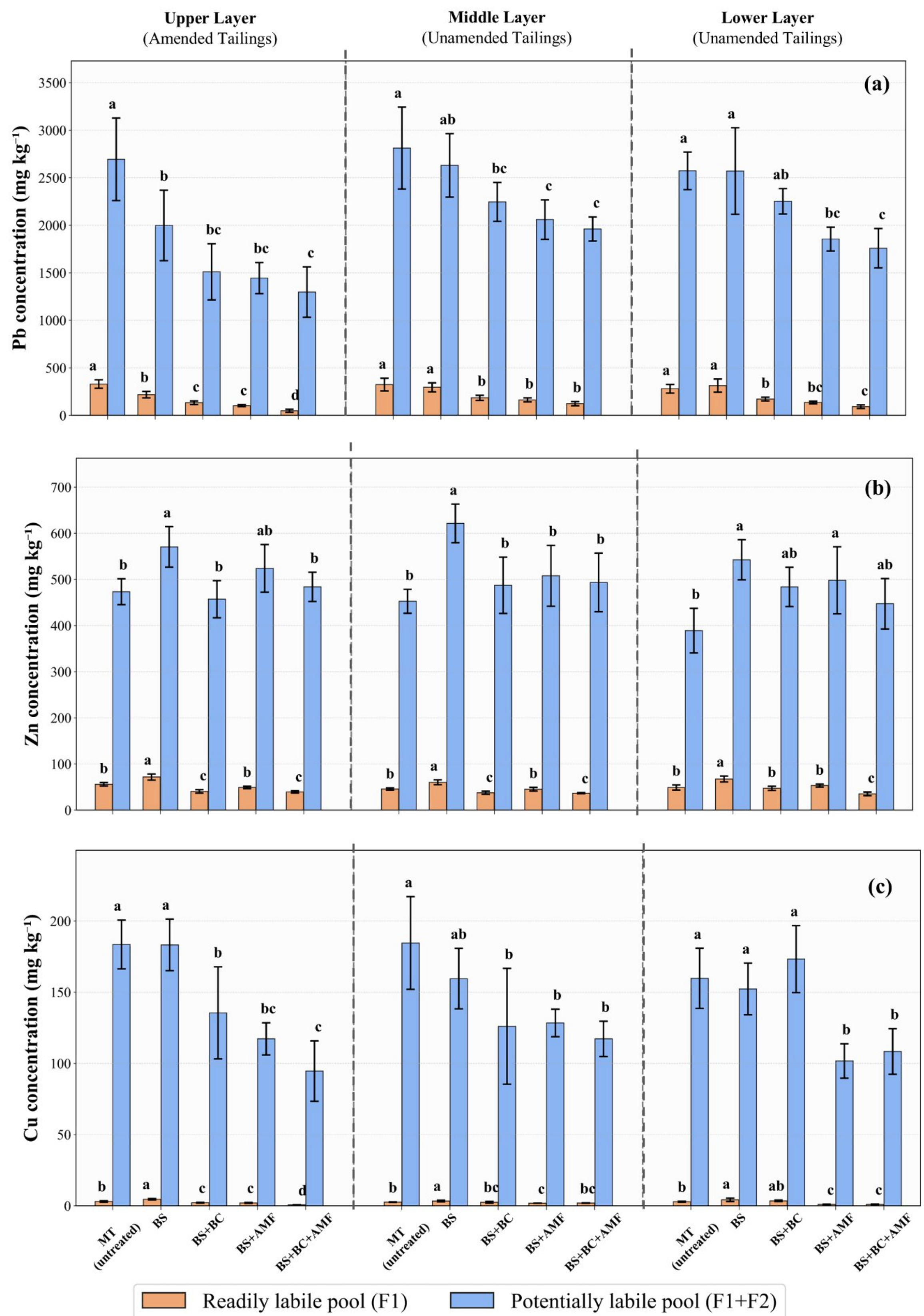
Upper Layer
(Amended Tailings)
Middle Layer
(Unamended Tailings)
Lower Layer
(Unamended Tailings)
(a)
(b)
(c)
Pb concentration (mg kg⁻¹)
Zn concentration (mg kg⁻¹)
Cu concentration (mg kg⁻¹)
MT (untreated)
BS
BS+BC
BS+AMF
BS+BC+AMF
Readily labile pool (F1)
Potentially labile pool (F1+F2)

Figure S5. Readily and potentially labile Pb, Zn, and Cu pools across tailings profile layers after amendment-assisted revegetation. Bars show the concentrations of **(a)** Pb, **(b)** Zn, and **(c)** Cu in the operationally defined readily labile pool (F1) and potentially labile pool (F1+F2) across treatments and profile depths. The upper layer represents amended tailings, whereas the middle and lower layers represent unamended tailings. Values are means ± SD (n = 5). Different letters above bars indicate significant differences among treatments within each tailings profile layer and metal pool based on Tukey's HSD post hoc test following one-way ANOVA (p < 0.05).

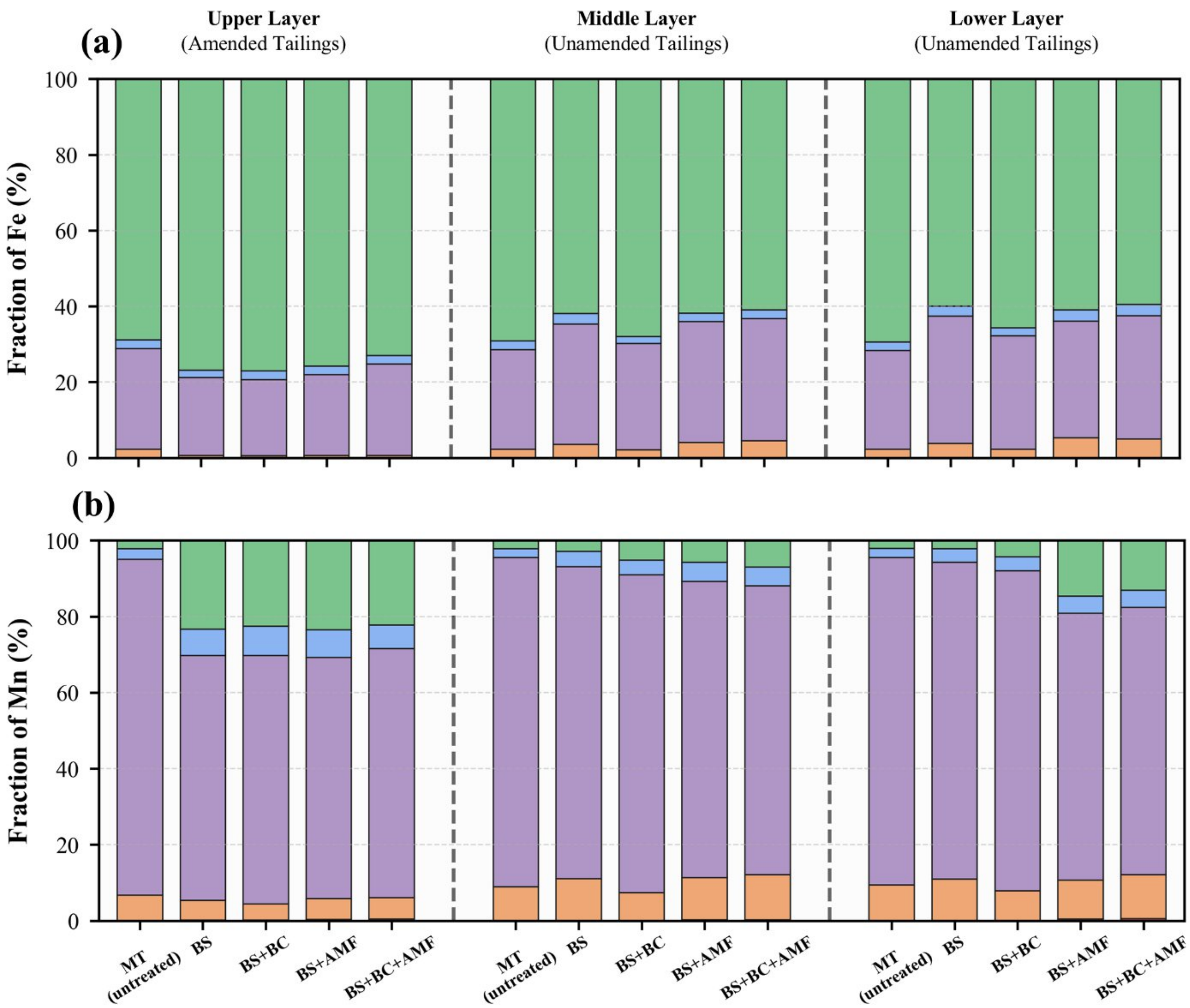

Figure S6. Fe and Mn fractionation across tailings layers after seven years of amendment-assisted revegetation. Stacked bars show the mean percentage distribution of **(a)** Fe and **(b)** Mn among sequential extraction fractions in the upper amended tailings layer and the middle and lower unamended tailings layers. Values are means (n = 5). Fractions are operationally defined

as F1, exchangeable; F2, carbonate-associated; F3, Fe/Mn oxide-associated; F4, organic/sulfide-associated; and F5, residual/non-silicate-bound.

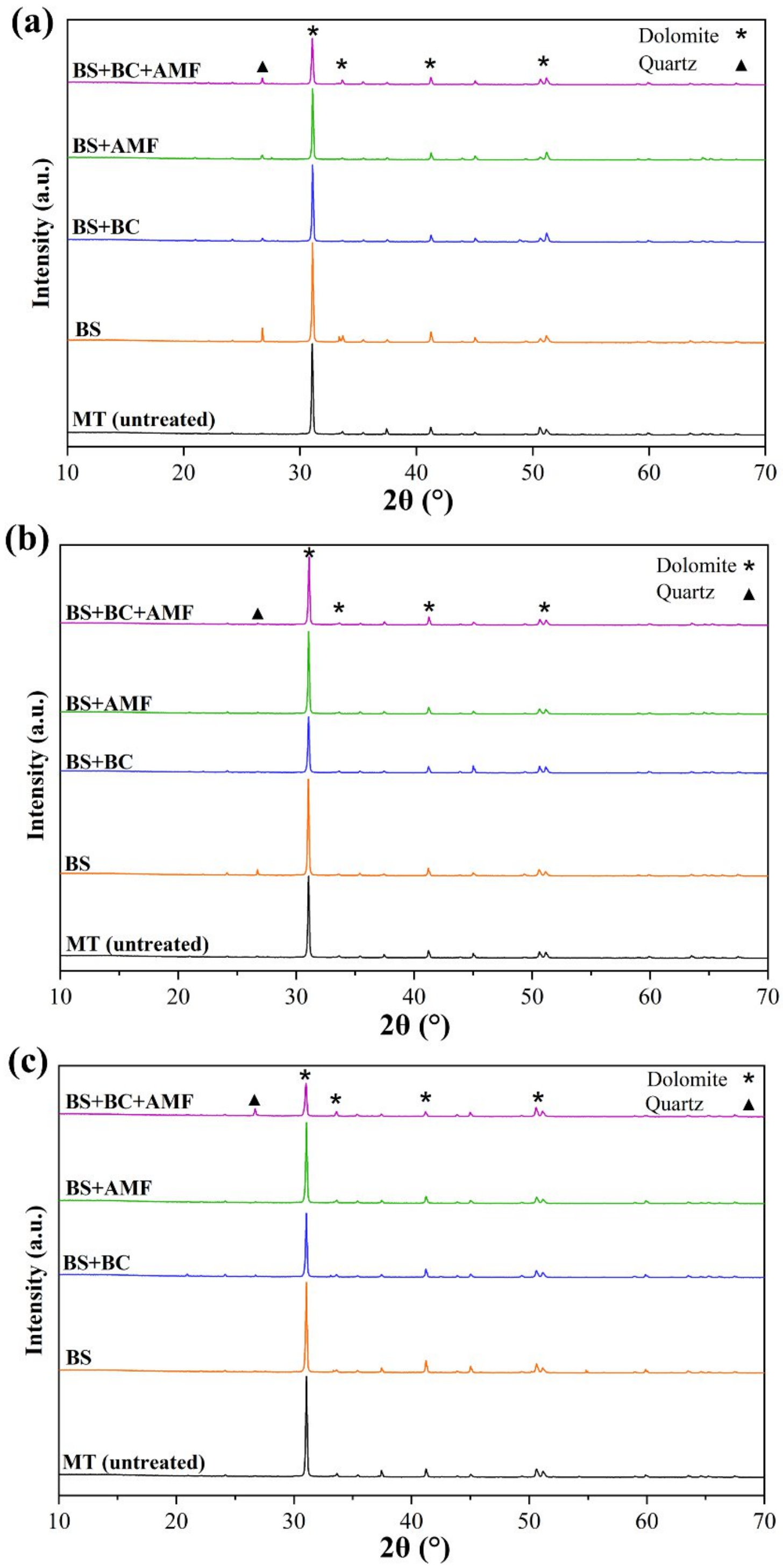

Figure S7. X-ray diffraction (XRD) patterns of tailings layers after seven years of amendment-assisted revegetation. Representative XRD patterns are shown for **(a)** upper amended tailings layer, **(b)** middle unamended tailings layer, and **(c)** lower unamended tailings layer.

References

1. Shrivastava, A.; Gupta, V. B., Methods for the determination of limit of detection and limit of quantitation of the analytical methods. *Chron. Young Sci* **2011,** *2* (1), 21-25.
